# Synaptic and mitochondrial mechanisms behind alcohol-induced imbalance of excitatory/inhibitory synaptic activity and associated cognitive and behavioral abnormalities

**DOI:** 10.1038/s41398-024-02748-8

**Published:** 2024-01-22

**Authors:** Thiago Arzua, Yasheng Yan, Xiaojie Liu, Ranjan K. Dash, Qing-Song Liu, Xiaowen Bai

**Affiliations:** 1 Department of Cell Biology, Neurobiology & Anatomy, Medical College of Wisconsin, Milwaukee, WI 53226 USA; 2 Department of Pharmacology, Medical College of Wisconsin, Milwaukee, 53226 WI USA; 3 Department of Biomedical Engineering, Medical College of Wisconsin, Milwaukee, 53226 WI USA

**Keywords:** Molecular neuroscience, Diseases

## Abstract

Alcohol consumption during pregnancy can significantly impact the brain development of the fetus, leading to long-term cognitive and behavioral problems. However, the underlying mechanisms are not well understood. In this study, we investigated the acute and chronic effects of binge-like alcohol exposure during the third trimester equivalent in postnatal day 7 (P7) mice on brain cell viability, synapse activity, cognitive and behavioral performance, and gene expression profiles at P60. Our results showed that alcohol exposure caused neuroapoptosis in P7 mouse brains immediately after a 6-hour exposure. In addition, P60 mice exposed to alcohol during P7 displayed impaired learning and memory abilities and anxiety-like behaviors. Electrophysiological analysis of hippocampal neurons revealed an excitatory/inhibitory imbalance in alcohol-treated P60 mice compared to controls, with decreased excitation and increased inhibition. Furthermore, our bioinformatic analysis of 376 dysregulated genes in P60 mouse brains following alcohol exposure identified 50 synapse-related and 23 mitochondria-related genes. These genes encoded proteins located in various parts of the synapse, synaptic cleft, extra-synaptic space, synaptic membranes, or mitochondria, and were associated with different biological processes and functions, including the regulation of synaptic transmission, transport, synaptic vesicle cycle, metabolism, synaptogenesis, mitochondrial activity, cognition, and behavior. The dysregulated synapse and mitochondrial genes were predicted to interact in overlapping networks. Our findings suggest that altered synaptic activities and signaling networks may contribute to alcohol-induced long-term cognitive and behavioral impairments in mice, providing new insights into the underlying synaptic and mitochondrial molecular mechanisms and potential neuroprotective strategies.

## Background

Prenatal alcohol exposure during pregnancy can lead to a group of long-lasting conditions known as Fetal Alcohol Spectrum Disorders (FASD) [1, 2]. The symptoms of FASD can vary from subtle behavior changes, such as impairments in cognition and behaviors, to significant morphological abnormalities, such as ethanol-induced ventriculomegaly [3–5]. The estimated prevalence of FASD is between 1–5% in the USA and Western Europe. Despite various public health campaigns worldwide, approximately 1 in 10–20 pregnant women in the USA and 50% in some parts of Europe still report consuming ethanol during pregnancy [6]. Sociocultural and economic factors, including a high number of unplanned pregnancies [7] (up to 50% of all pregnancies in the USA) and alcohol use disorder (AUD), make it challenging to prevent alcohol consumption through education alone, and it is likely that the actual prevalence of FASD is higher than estimated [8, 9]. Unfortunately, FASD currently has no cure or specific therapy, apart from palliative care [10]. Ethanol-induced developmental neurotoxicity (EIDN) manifests as key cognitive impairments and behavioral problems throughout life, but the underlying mechanisms still largely unknown. Recent studies suggest that excitatory/inhibitory (E/I) imbalances and abnormal mitochondrial activity are critically important for neurodevelopmental disorders and neurodegeneration (e.g., Autism Spectrum Disorder, Timothy syndrome, general anesthesia neurotoxicity, and Alzheimer’s Disease) associated with abnormal cognition and behaviors [11–17].

Maintaining a balance between excitation and inhibition is critical for the formation and function of synaptic circuits, which are essential for normal behavior, cognition, and memory under physiological conditions [18]. This balance is regulated both at the level of individual neurons, by controlling the number of specific glutamatergic excitatory and γ-aminobutyric acid (GABA) inhibitory neurons, and at the network level, by regulating communication between specific circuits [19, 20]. Much of the literature regarding ethanol and E/I imbalances has focused on adult modes of AUD, but interestingly, several studies have shown that this is especially important during development. For example, Skorput et al. found that exposure of fetal mice to ethanol during embryonic day 13.5–16.5 resulted in a shift in E/I balance towards inhibition [21]. Similarly, studies have found that the specific subunit GluN2B of the glutamatergic N-methyl-D-aspartate (NMDA) receptor is specifically affected by early exposure to ethanol [22]. Given that ethanol can act in both excitatory and inhibitory synapses, and that the maintenance of that balance is particularly important in early development, it is crucial to understand the underlying molecular mechanisms by which EIDN relates to E/I imbalance.

Furthermore, there are key links between E/I balance and the bioenergetic modulation of neurons through the mitochondria [23]. Mitochondrial health is essential for the proper functioning of the central nervous system. Mitochondria are involved in various cellular processes, such as production of ATP and regulation of intracellular calcium (Ca^2+^) homeostasis. Neurons have extremely high energy demand, consuming 80% of brain energy. Mitochondria play a central role in the complex behavior of neurons, including establishing membrane excitability, forming synapse, and regulating neurotransmission and plasticity [24]. Changes in mitochondrial dynamics, function, and molecular signaling have been linked to neuronal dysfunction, impaired synaptic homeostasis, strength, and plasticity, and a range of neurological diseases, such as intellectual disabilities [25]. We and others have shown that ethanol exposure impaired acute impaired mitochondrial bioenergetics and changes in mitochondrial ultrastructure [26–29]. Earlier studies started unveiling the role of ethanol-induced excitotoxicity, that is toxicity linked to ethanol exposure and elevation of extracellular glutamate in neurons, by linking that to ATP depletion and intracellular Ca^2+^ dynamics [30]. This has been expanded recently by demonstration that ethanol exposure during adolescence affects both mitochondrial permeability, and synaptic function – leading to impairments lasting into adulthood [31]. In models of AUD, studies have also found that the medial prefrontal cortex (mPFC) in mice, a region containing more mitochondria than other cortical areas, is susceptible to ethanol-induced dysregulation of neuronal bioenergetics [32]. In other forms of neurodegeneration, such as Alzheimer’s and Parkinson’s, there is also evidence of direct connections between mitochondrial health, Ca^2+^ handling, and excitotoxicity – what Verma et al termed the triad in synaptic neurodegeneration [33]. Nevertheless, whether developmental ethanol exposure influences long-term mitochondrial gene transcriptomics remains unknown. Additionally, no studies have investigated the long-term interrelationship between mitochondrial and synapse molecular signaling, E/I imbalance, and cognitive and behavioral impairment in EIDN [33].

Therefore, this study aims to investigate the association between E/I imbalance, cognitive dysfunction, behavioral deficits, and the long-term changes in mitochondrial and synaptic gene profiles and signaling networks in postnatal day 60 (P60) adult mice that were exposed to ethanol during development. Specifically, we used a mouse model of binge drinking during the third trimester-equivalent P7 to examine EIDN and its underlying cellular and molecular mechanisms. First, we investigated the ethanol-induced acute neuroapoptosis in P7 mice and the long-term spatial learning/memory and anxiety levels of adult mice exposed at the same developmental time point. Next, we characterized the E/I imbalance present in the hippocampi of adult mice that were developmentally exposed to ethanol. Finally, we used transcriptomic data from a previous study utilizing the same model of EIDN [34] to profile the dysregulation of synaptic- and mitochondria-related genes and their respective computationally predicted pathways related to neuronal function, synaptic activities, developmental disorders, cognitive and behavioral problems.

## Methods

### Ethanol exposure

**Fig. 1:**
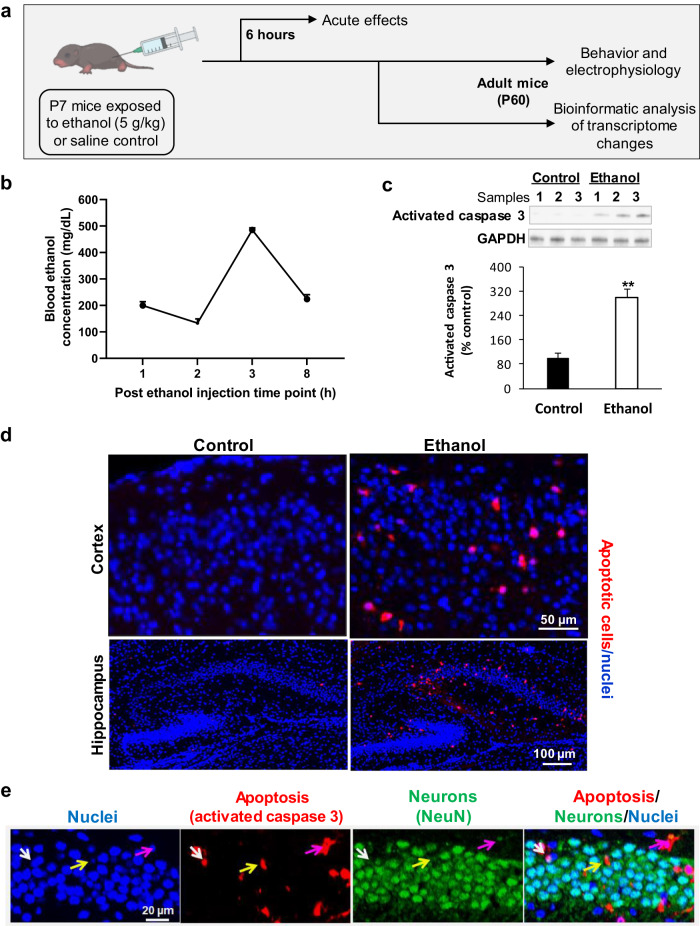
Ethanol induced neuroapoptosis in postnatal day 7 (P7) mouse brains. **a** Schematic representation of the model used in the study, depicting P7 mice injected with 5 g/kg of ethanol and subsequent experiments. **b** Blood ethanol concentration within 8 hours following the first ethanol injection in mice. *n* = 3 to 4. **c** Western blot analysis showed that ethanol exposure at P7 increased the expression of activated caspase 3, an apoptotic marker, in mouse brains.**p < 0.01, *n* = 3. **d** Immunofluorescence staining and imaging revealed activated caspase 3-positive apoptotic cells in the cortex and hippocampal tissue of mice. Blue represents cell nuclei and red represents activated caspase 3-positive apoptotic cells. Scale bar = 50 or 100 µm. **e** Ethanol exposure resulted in apoptosis in neurons in the hippocampus, where the activated caspase 3-positive apoptotic signals (red) were located in neurons positive for neuronal nuclear antigen (NeuN), a neuron marker (green). Blue represents cell nuclei stained with Hoechst 33342. Three representative apoptotic neurons are indicated by white, yellow, and pink arrows. Scale bar = 20 µm.

All animal experiments described were approved by the Institutional Animal Care and Use Committee at the Medical College of Wisconsin. C57BL/6 mice (Jackson Laboratories, Bar Harbor, ME, USA) received an ethanol exposure on P7 when the developing brain is most vulnerable to ethanol, and the equivalent of a third-trimester fetal in humans [35, 36]. Both male and female mice were included in the studies and randomly distributed into ethanol or control groups. The mice were exposed to the ethanol using the approaches as described [36, 37]. Mice were randomly assigned to either ethanol or control saline group. Ethanol anhydrous (MilliporeSigma, Burlington, MA, USA) was freshly dissolved in saline to a final concentration of 20% weight/volume. Mice were injected subcutaneously with 2.5 g/kg ethanol or saline at 0 h, and again after 2 h (total 5 g/kg ethanol injected) to mimic binge-like ethanol exposure. This regimen was chosen based on similar rodent studies that have established the apoptotic effects of a single day of exposure [36]. Figure 1a depicted the experimental design of mouse studies. Brain tissue was collected 6 h after the initial injection for immunofluorescence imaging and western blot analysis of apoptosis, or at P60 for electrophysiological assays. Mouse blood ethanol concentrations (BECs) were collected within 8 h after the injection of ethanol and quantified using Ethanol Assay Kit (Colorimetric) (Cell Biolabs, INC, San Diego, CA, USA). Histological and Western blot assays were performed at P6 mice, and bioinformatical analysis of gene expression profiles, electrophysical analysis, and cognitive and behavioral tests were conducted using P60 mice. Experiments were analyzed blindly when possible.

### Immunofluorescence staining

In brief, 4 µm-thick sagittal sections were obtained from P7 paraffin-embedded brain tissue blocks. The sections were then deparaffinized, hydrated, and subjected to antigen retrieval and washes with phosphate-buffered saline (PBS) containing 0.5% Triton X-100 (MilliporeSigma), as previously described [38]. To investigate whether neurons undergo apoptosis following ethanol exposure, the sections were co-stained with rabbit anti-activated caspase 3 (apoptosis marker; Cell Signaling, Danvers, MA, USA; #9664) and either mouse anti-neuronal nuclear antigen (NeuN: neuron marker; MilliporeSigma, MAB377) for 1 h at 37 °C [26]. Following three washes, the slides were incubated with Alexa Fluor 488-conjugated donkey anti-mouse IgG or goat IgG along with Alexa Fluor 594-conjugated donkey anti-rabbit (Thermo Fisher Scientific, Waltham, MA, USA) for 45 min at 37 °C. After three more washes with PBS, the cellular nuclei were stained with Hoechst 33342 (Thermo Fisher Scientific). The stained sections were imaged using an Olympus Fluorescent Slide Scanner (Olympus, Shinjuku City, Tokyo, Japan).

### Western blot

P7 brain tissues were harvested and homogenized in RIPA lysis buffer (Cell Signaling, Danvers, MA, USA) supplemented with a cocktail of phosphatase and protease inhibitors (Roche Diagnostics, Barrington, IL) [39]. The lysates were centrifuged at 10,000 × *g* for 10 min at 4 °C and the supernatants were collected. The protein concentration in each sample was determined using a DC Protein Assay Reagents Package kit (Bio-Rad, Hercules, CA, USA). Equal amounts of protein (25 μg) were loaded per lane for sodium dodecyl sulfate polyacrylamide gel electrophoresis (SDS-PAGE) and transferred to a nitrocellulose membrane. The membrane was blocked with blocking buffer (ThermoFisher Scientific) and incubated overnight at 4 °C with primary antibodies, rabbit anti-activated caspase 3 (Cell Signaling, Danvers, MA, USA; #9664) and rabbit anti-β-actin (Santa Cruz, Dallas, TX, USA; sc-47778). After washing with Tris-buffered saline containing 0.1% Tween 20, the membrane was incubated with horseradish peroxidase-conjugated secondary antibodies (Cell Signaling) for one hour at room temperature and then with chemiluminescence detection reagent (Cell Signaling). The labeled proteins were visualized using a Chemidoc imaging system (Bio-Rad) and the optical densities of protein signals were quantified using ImageJ software. The abundance of protein level was normalized to an internal control of β-actin.

### Open field test

To evaluate the anxiety-like behavior of mice, we utilized the open field test, which assesses both exploratory and locomotor activity [40, 41]. After acclimating in a separate room for an hour, each mouse was placed in the center of a circular chamber with a radius of 44 cm and allowed to explore freely for 10 min, while their movements were recorded via video. We analyzed the distance traveled and immobile time in the chamber using the advanced tracking software EthoVision XT.

### Morris water maze

To evaluate spatial learning and memory of P60 mice that received ethanol exposure at P7, we utilized the Morris water maze as described previously [42]. The maze consisted of a circular polypropylene pool (100 cm in diameter and 20 cm in height) filled with opaque water containing non-toxic white paint. Four designated points on the rim of the pool (north, east, south, and west) divided the pool into four quadrants (NE, NW, SW, and SE). A platform (8 × 8 cm) was placed at the center of the SE quadrant, submerged ~2 cm below the water’s surface. Each mouse was tracked via EthoVision XT (Noldus Information Technology, Washington, USA) starting from a random start point until it reached the platform, or after 1 min. If the mouse was unable to find the platform within 1 min, the investigator guided it to reach the platform. Trials were repeated 4 times per day, with 5-min intervals, for 5 consecutive days. The latency to reach the platform was measured as an indicator of spatial learning. On the 6th day, the platform was removed, the mouse was placed in a new start point in the pool and was allowed to swim for one minute while being tracked. The latency for the mice to find the zone where the platform was placed during the learning test was recorded as a measure of spatial memory.

### Electrophysiological assays

P7 control and ethanol-exposed P60 mice were decapitated, and their hippocampi were dissected and embedded in low-gelling-point agarose (3%, MilliporeSigma). Transverse hippocampal slices were then cut at a thickness of 200 μm using a vibrating slicer (Leica VT1200s, Nussloch, Germany) in a choline-based solution containing 110 choline chloride, 2.5 KCl, 1.25 NaH_2_PO_4_, 0.5 CaCl_2_, 7 MgSO_4_, 26 NaHCO_3_, 25 glucose, 11.6 sodium ascorbate, and 3.1 sodium pyruvate [43]. The slices were subsequently transferred to and stored in artificial cerebrospinal fluid (ACSF) containing 119 NaCl, 3 KCl, 2 CaCl_2_, 1 MgCl_2_, 1.25 NaH_2_PO_4_, 23 NaHCO_3_, and 10 glucose at room temperature for at least 30 min before use. All solutions were saturated with 95% O_2_ and 5% CO_2_.

Whole-cell voltage-clamp recordings were conducted using patch clamp amplifiers (Multiclamp 700B) under infrared-differential contrast interference microscopy. Data acquisition and analysis were performed using digitizers (DigiData 1440A and 1550B) and analysis software pClamp 10 (Molecular Devices). Signals were filtered at 2 kHz and sampled at 10 kHz. For recording spontaneous excitatory postsynaptic currents (sEPSCs), we used freshly prepared picrotoxin (50 μM), a GABA-A receptor blocker, dissolved in the ACSF through sonication for approximately 10 min. Hippocampal CA1 pyramidal neurons were voltage clamped at −70 mV with an internal solution consisting of 140 K-gluconate, 5 KCl, 2 MgCl_2_, 10 HEPES, 0.2 EGTA, 4 Mg-ATP, 0.3 Na_2_GTP, and 10 Na_2_-phosphocreatine at pH 7.2 (with KOH). To record spontaneous inhibitory postsynaptic currents (sIPSCs), we used glutamate receptor antagonists 6-cyano-7-nitroquinoxaline-2,3-dione (CNQX, 10 µM) and D-2-amino-5-phosphonovaleric acid (D-AP-5, 20 µM) throughout the experiments. Hippocampal CA1 pyramidal neurons were voltage clamped at −70 mV with an internal solution consisting of 80 K-gluconate, 60 KCl, 10 HEPES, 0.2 EGTA, 2 MgCl_2_, 2 Mg-ATP, 0.3 Na_2_GTP, and 10 Na_2_-phosphocreatine at pH 7.2 (with KOH). We monitored the series resistance (15-30 MΩ) throughout the recordings and discarded any data if the resistance changed by more than 20%. We obtained CNQX, D-AP5, and picrotoxin from Tocris Bioscience (Ellisville, MO, USA). All other commonly used chemicals were purchased from MilliporeSigma. Mini-analysis software (Synaptosoft, Decatur, GA, USA) was used to analyze the sEPSC and sIPSC data. Cumulative probability plots were used to analyze the sEPSC and sIPSC data [44].

### Microarray assay of messenger RNA (mRNA) profiling

After conducting a thorough literature review, we came across a published study with publicly available transcriptomic datasets and the same model as our experiments (P7 mice injected with 2 × 2.5 g/kg of ethanol and analyzed at P60) [34]. The study by Kleiber et al exposed mice to ethanol during the first (embryonic day, E8/11), second (E14/5), and third (P4/7) trimesters of human pregnancies, and through microarray analysis, identified transcriptomic changes that persisted in P60 mice. The findings revealed that ethanol disrupts the brain transcriptome at every stage, but each stage leads to a unique “footprint” of dysregulated genes [34]. With this information, we decided to analyze the same dataset, focusing on the genes from the third-trimester group, with the mechanistic premise of examining synaptic and mitochondrial-related genes. Subsequently, we isolated these genes and performed the bioinformatic analyses as detailed below.

### Bioinformatic analysis of ethanol-induced dysregulated synaptic genes and mitochondrial genes and the related pathways/functions

To better understand the molecular mechanisms behind ethanol-induced long-term E/I imbalance and cognitive and behavioral impairments, we conducted bioinformatic analyses on 376 dysregulated mRNAs induced by human third-trimester-equivalent ethanol exposure in P60 mouse brains, identified at a fold-change cutoff of 1.2 and *p* < 0.05 [34] (see Table S1). We utilized several bioinformatic analysis tools, including Ingenuity Pathway Analysis (IPA), Synaptic Ontology (SynGO), mitoXplorer, and Metascape databases, to investigate the dysregulated genes and their related pathways/functions as described below.

To investigate the contribution of synaptic gene signaling to ethanol neurotoxicity, we identified ethanol-induced dysregulated synapse-related genes using the SynGO database (https://www.syngoportal.org) [45], which provides annotations based solely on published experimental evidence. We used a brain-expressed background gene set to identify enriched synaptic components from the ethanol-induced dysregulated genes. The SynGo database includes information on the synaptic localization and function of approximately 1,112 synaptic genes. To explore the contribution of mitochondrial gene signaling in ethanol neurotoxicity, we identified ethanol-induced dysregulated mitochondria-related genes through the mitoXplorer database. MitoXplorer is a web-based platform that enables analysis and visualization of genes that are involved in various mitochondrial processes, such as energy production, metabolism, and signaling (http://mitoxplorer.ibdm.univ-mrs.fr/about.html) [46]. To further investigate the cellular function/pathway and diseases associated with ethanol-induced dysregulated synapse and mitochondria-related genes, we performed enrichment pathway analysis of differentially expressed synaptic genes using Metascape (http://metascape.org) as previously described [47].

Next, we used the IPA software (Qiagen) to analyze the signaling/pathways of developmental ethanol exposure-induced dysregulated synaptic and mitochondrial genes in adult P60 mouse brains, as well as their association with cognitive dysfunction and neurological diseases. IPA predicts disease mechanisms and canonical physiological signaling pathways based on differentially expressed genes between different conditions [48]. The signaling pathways and networks of the dysregulated genes were analyzed based on the known individual gene’s participation in established pathways from the literature included in the IPA database. We obtained a collection of predictions regarding roles of the genes in the central nervous system development and function, behavior, and neurological diseases (with Fisher’s exact test *p* < 0.05 calculated in the IPA database).

### Statistic analysis

All data were presented as mean ± standard error (SE) of the mean. Sample size was determined based on pilot data from our laboratory and previous similar studies. For BEC and apoptosis analysis, we used *n* = 3–4 per group, *n* = 7–10 per group for behavior tests and electrophysiology analyses. The data of the ethanol-induced dysregulated genes in P60 mouse brain were obtained from 6 mice per groups. Statistical analysis was performed using unpaired Student’s t-test in GraphPad Prism (version 9.0) to compare the control and ethanol treatment groups. The level of statistical significance was set at *p* < 0.05. The normal distribution of data was analyzed using either Shapiro–Wilk or Kolmogorov–Smirnov normality tests, as appropriate for the specific statistical analysis.

## Results

### Ethanol exposure induces acute neuroapoptosis in neonatal mouse brains

An established regimen of subcutaneous injections of 2 × 2.5 g/kg of ethanol was administered to P7 mice, as shown in Fig. 1a. This injection resulted in BEC of 200, 133, 485, and 223 mg/dL at 1, 2, 3 and 8 hours, respectively, with the average peak BEC occurring at 3 hours after the first dose of ethanol (Fig.1b). These results are consistent with previous reports using the same model, all of which found increased apoptosis in different brain regions with a single exposure [37, 49, 50]. Western blot analysis and immunofluorescence staining of brain tissue isolated 6 h after the first injection revealed a significant and dramatic increase in apoptosis, as measured by the expression of activated caspase 3 (Fig. 1c). Immunofluorescence staining specifically highlighted the apoptotic effects of ethanol in the cortex and hippocampus (Fig. 1d). To further investigate the apoptotic effect on hippocampal neurons, we co-stained slices using anti-activated caspase 3 and anti-neuronal nuclear antigen (NeuN) antibodies. The staining showed colocalization between both markers, indicating an increase in neuroapoptosis in the hippocampi of exposed mice (Fig. 1e). These findings demonstrate the acute apoptotic effect of binge drinking-like exposure to ethanol on the developing brain.

### Developmental ethanol exposure leads to anxiety-like behavior and impaired learning in adult mice

**Fig. 2:**
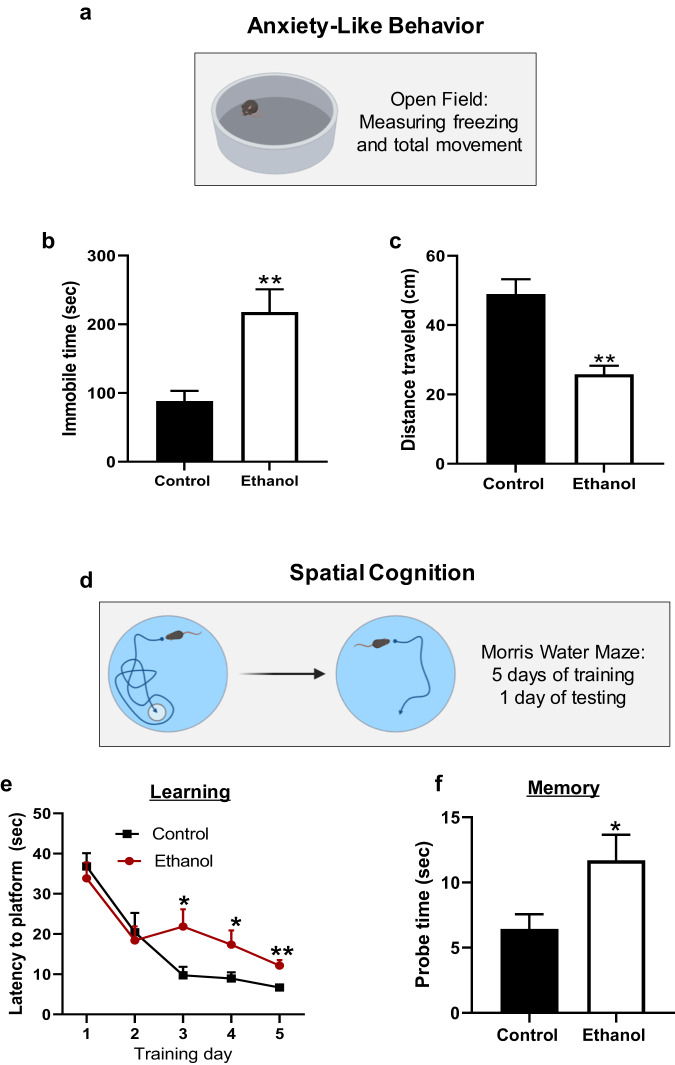
Neonatal ethanol exposure resulted in anxiety-like behaviors and impaired spatial learning abilities in P60 mice. **a**–**c** Open field tests revealed that P60 mice exposed to ethanol at P7 spent more time in periods of total immobile and had less distance traveled, indicating an increase in anxiety-like behavior, given that no specific motor disability was noted during the Morris water maze test. **d**–**f** A 5-day learning test and one-day memory test were conducted using the Morris water maze. The mice treated with ethanol took longer (escape latency) to find the platform than the control mice on days 3–5 during the learning test and on day 6 during the memory test. *n* = 7. *p < 0.05, **p < 0.01 vs. control.

We conducted studies to investigate the long-term effects of developmental ethanol exposure on behavior and cognition in mice. We utilized an open field test to evaluate anxiety-like behavior in P60 adult mice exposed to ethanol at P7 (Fig. 2a). Our findings revealed that these mice displayed more immobile behavior and decreased overall locomotion, consistent with previous studies examining anxiety-like behavior in mice [51, 52] (Fig. 2b). Furthermore, the ethanol-exposed mice traveled a significantly shorter distance compared to the control mice (Fig. 2c). To measure spatial cognition, we used the Morris water maze and trained mice for 5 days (learning) before testing them on day 6 (memory) (Fig. 2d). We observed significant impairments in the ability of ethanol-exposed mice to learn the location of the platform starting from the second day of training, which persisted throughout the training, as evidenced by increased latency to reach the platform (Fig. 2e). Interestingly, on the probe day when the platform was removed, the ethanol-exposed mice showed to have decreased memory of the platform location compared to the control mice (Fig. 2f). These findings suggest that developmental binge-like exposure to ethanol causes significant long-term changes in anxiety-like behavior and spatial cognition in adult mice.

### Ethanol exposure in neonatal mice causes the disruption of E/I balance in the hippocampal neurons of adult mice

**Fig. 3:**
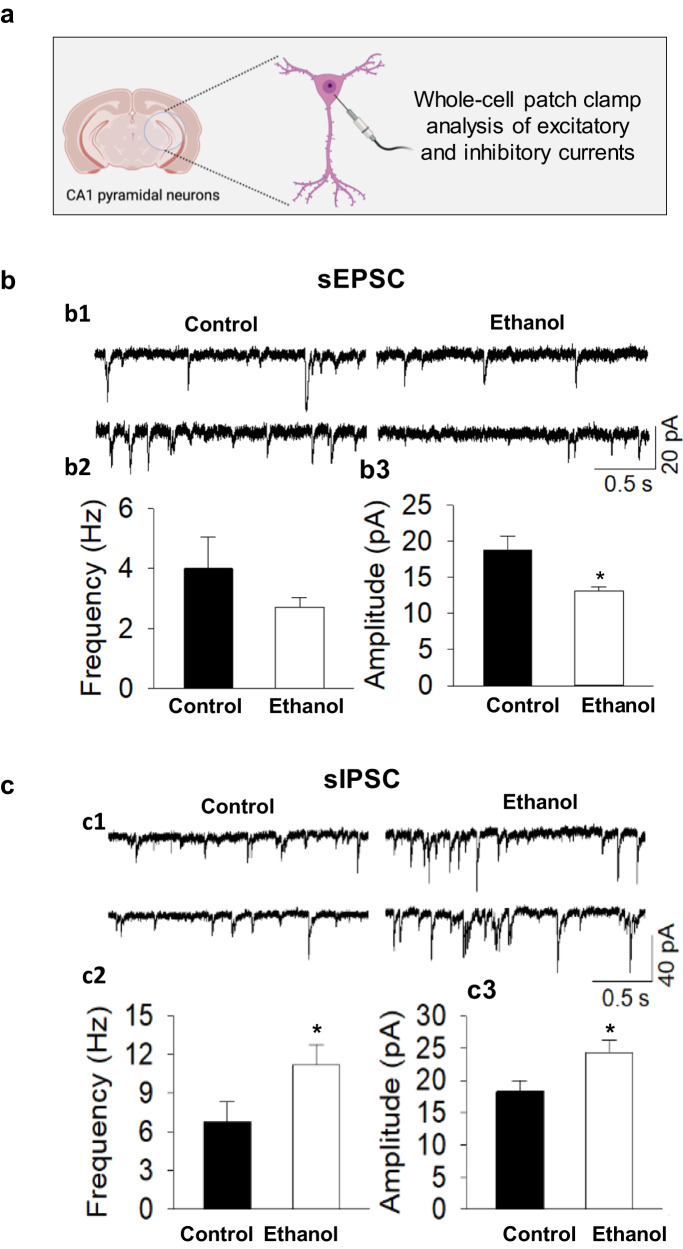
Ethanol caused long-term excitatory/inhibitory (E/I) imbalance in hippocampal CA1 pyramidal neurons in P60 mice subjected to ethanol exposure at P7. **a** Schematic representation of the whole-cell patch clamp electrophysiology performed in hippocampal slices. **b** Ethanol-treated mouse neurons showed a decrease in spontaneous excitatory postsynaptic currents (sEPSC) amplitude. **b1** Representative traces of sEPSCs recorded. **b2-3** Mean frequency and amplitude of sEPSCs. **c** Ethanol exposure resulted in increased spontaneous inhibitory postsynaptic currents (sIPSC) frequency and amplitude in neurons. **c1** Representative traces of sIPSCs recorded. **c2-3** Mean frequency and amplitude of sIPSCs. *n* = 7 to 10. *p < 0.05.

To investigate the synaptic mechanisms of ethanol-induced impairment of cognition, we analyzed the balance of E/I in neurons of the cornu ammonis 1 (CA1) region of the hippocampus. To accomplish this, we prepared hippocampal slices from P60 mice that had received either ethanol or saline injections at P7 and conducted whole-cell recordings in visually identified CA1 pyramidal neurons in the slices (Fig. 3a). We recorded spontaneous excitatory postsynaptic currents (sEPSCs) and spontaneous inhibitory postsynaptic currents (sIPSCs), as we have described [17, 53]. Our results indicated a significant decrease in the mean amplitude of sEPSCs in ethanol-treated mice (*p* < 0.05), while the mean frequency was not significantly affected (*p* = 0.262) (Fig. 3b). Conversely, there was a significant increase in the mean frequency (*p* < 0.05) and mean amplitude (*p* < 0.05) of sIPSCs in ethanol-treated mice compared to those in saline-treated mice (Fig. 3c). These findings suggest that P7 ethanol treatment leads to a decrease in excitatory transmission and an increase in inhibitory transmission in hippocampal CA1 pyramidal neurons. Such a shift in E/I balance is indicative of long-term synaptic dysfunction and disruption of normal synaptic plasticity, leading to impaired cognition in adult mice exposed to ethanol during development.

### Ethanol exposure during development induces abnormal expression of synaptic genes

**Fig. 4:**
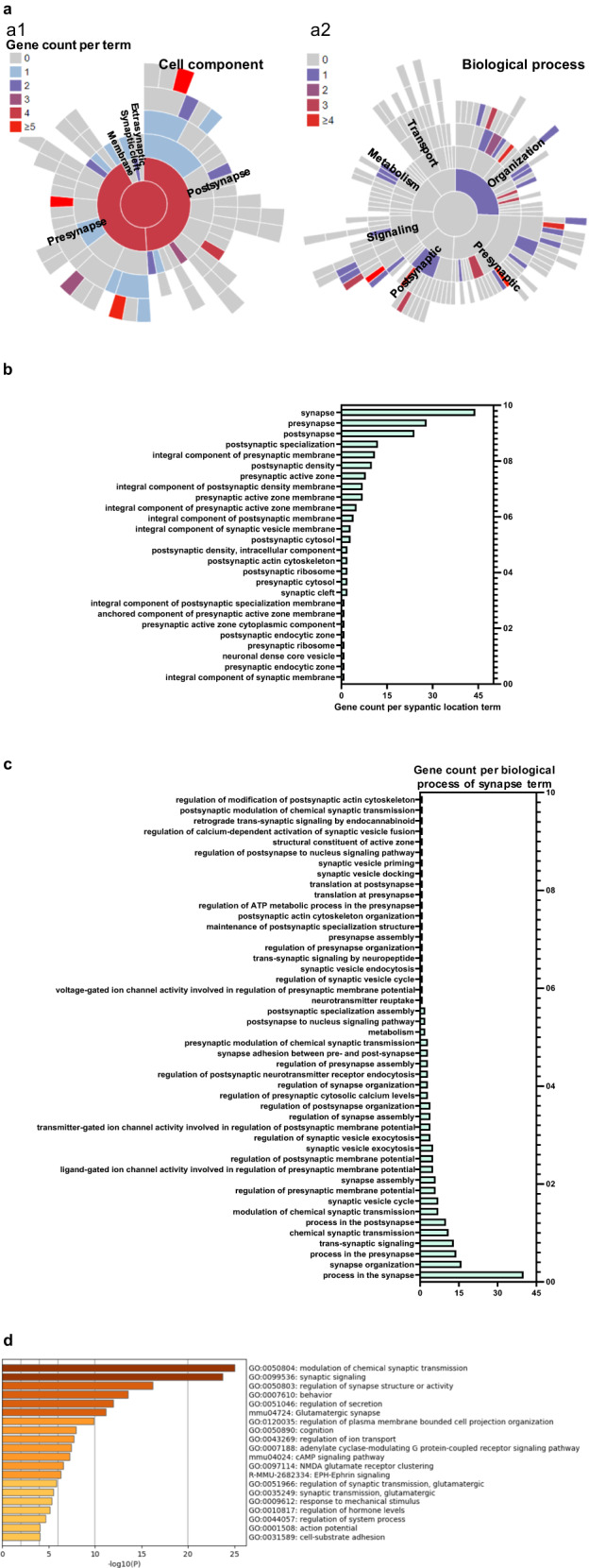
Developmental ethanol exposure dysregulated synaptic genes in P60 mouse brain tissue. **a** Bioinformatic analysis of 376 ethanol-induced differentially expressed genes (*n* = 6, fold change cutoff of 1.2 vs. control, *p* < 0.05) in P60 mouse brains was performed to identify dysregulated synaptic genes through the SynGO database. The analysis revealed 50 ethanol-induced dysregulated genes that were synaptic genes, of which 48 genes were downregulated and two genes were upregulated. The sunburst plot illustrates the synapse location (cell component for presynapse, postsynapse, synaptic cleft, extra-synaptic space, or synaptic membranes) and functions (biological process related to metabolism, transport, synapse organization, synaptic signaling, presynapse, and postsynapse) of these 50 synaptic genes. The different colors represent the gene counts per term of synaptic location (**a1**) or each function of the synapse (**a2**). Additional information on the synapse location and function of these 50 genes is provided in Table 1. **b** The horizontal bar graph shows the gene count per synaptic cellular component, as illustrated in (**a1**). **c** The horizontal bar graph shows the gene count per synaptic biological process, as shown in (**a**-**b**). **d** Gene ontology (GO) analysis of pathway and process of ethanol-induced dysregulated synaptic genes was conducted using the Metascape bioinformatic tool. The bar graph illustrates the enriched terms of signaling, neuronal and synaptic activities, and cognition and behavior across the synaptic gene lists.

To understand the mechanisms underlying ethanol-induced synaptic dysfunction, impaired cognition, and anxiety-like behavior, we analyzed a publicly available transcriptomic dataset (Table S1) [34]. Kleiber et al. found that third trimester-equivalent ethanol exposure led to a unique fingerprint of dysregulated brain genes [34]. We reanalyzed their data to identify ethanol-induced abnormally expressed synaptic genes that are directly involved in synapse function and structure, as well as downstream circuits that support learning, memory, and behavior. Out of the 376 ethanol-dysregulated genes in P60 mouse brains, we identified 50 synaptic genes (48 downregulated and 2 upregulated) from the SynGO database. A summary of the synaptic genes dysregulated by ethanol is listed in Table 1. Most of these gene-encoded proteins were located in the presynaptic or postsynaptic regions, with some found in the synaptic cleft, extracellularly, and membrane (Fig. 4a1, b). Most of these ethanol-induced dysregulated synaptic genes participate in the synapse organization and trans-synaptic signaling, along with a few genes involved in various other synaptic activities and signaling. These include the regulation of ion channel activity, synaptic vesicle exocytosis, calcium signaling, presynapse assembly, and postsynapse organization (Fig. 4a2, c). By using Gene Ontology (GO) term enrichment analysis, a bioinformatics tool, we identified that the synaptic genes dysregulated by ethanol exposure were associated with several biological pathways and processes, including modulation of chemical synaptic transmission, synaptic signaling, regulation of synapse structure or activity, regulation of secretion, regulation of plasma membrane-bound cell projection organization, regulation of ion transport, cognition, and behavior (Fig. 4d).

**Table 1:** Ethanol-induced dysregulated synaptic genes and their locations in cellular component and involved biology processes.

Gene name	Location of cellular component	Related to biology process of synapse	Expression (ethanol vs. control)
Adgrl2	Integral component of postsynaptic membrane	Synapse organization	Down
Alk	Postsynapse	Trans-synaptic signaling by neuropeptide	Down
Apba1	Presynaptic active zone membrane	Presynaptic modulation of chemical synaptic transmission	Down
Apoe	synaptic cleft	Regulation of synapse organization	Down
Cacna1c	Integral component of postsynaptic membrane		Down
Cbln2		Modulation of chemical synaptic transmission, regulation of presynapse assembly	Down
Chrm1	Integral component of postsynaptic membrane	Regulation of postsynaptic membrane potential, postsynaptic modulation of chemical synaptic transmission	Down
Cnr1	Integral component of presynaptic membrane	Retrograde trans-synaptic signaling by endocannabinoid, regulation of presynaptic cytosolic calcium levels, regulation of ATP metabolic process in the presynapse	Down
Cpeb3	Postsynapse		Down
Dmd	Postsynaptic specialization		Down
Efna5		Synapse adhesion between pre- and postsynapse	Down
Efnb2	Integral component of presynaptic membrane, integral component of postsynaptic density membrane	Presynapse assembly, regulation of postsynaptic membrane neurotransmitter receptor levels, regulation of postsynaptic neurotransmitter receptor endocytosis	Down
Ephb1		Modulation of chemical synaptic transmission	Down
Fga	Synapse		Down
Grid1	Integral component of postsynaptic membrane, integral component of postsynaptic density membrane	Regulation of postsynapse organization	Down
Grik1	Integral component of presynaptic membrane	Ligand-gated ion channel activity involved in regulation of presynaptic membrane potential, transmitter-gated ion channel activity involved in regulation of postsynaptic membrane potential	Down
Grik3		Ligand-gated ion channel activity involved in regulation of presynaptic membrane potential, transmitter-gated ion channel activity involved in regulation of postsynaptic membrane potential	Down
Grin2b	Integral component of presynaptic membrane, integral component of presynaptic active zone membrane	Ligand-gated ion channel activity involved in regulation of presynaptic membrane potential, transmitter-gated ion channel activity involved in regulation of postsynaptic membrane potential	Down
Grin2c		Transmitter-gated ion channel activity involved in regulation of postsynaptic membrane potential	Down
Grin2d	Integral component of presynaptic membrane, integral component of presynaptic active zone membrane	Ligand-gated ion channel activity involved in regulation of presynaptic membrane potential, transmitter-gated ion channel activity involved in regulation of postsynaptic membrane potential	Down
Grin3b	Integral component of presynaptic active zone membrane, integral component of postsynaptic density membrane	Ligand-gated ion channel activity involved in regulation of presynaptic membrane potential	Down
Grk3	Postsynaptic density, presynapse		Down
Grm4	Integral component of presynaptic active zone membrane	Presynaptic modulation of chemical synaptic transmission	Down
Hap1	Presynaptic cytosol, postsynaptic cytosol	Regulation of postsynaptic neurotransmitter receptor endocytosis	Up
Htr1a	Integral component of presynaptic membrane	Presynaptic modulation of chemical synaptic transmission	Down
Htr1d	Neuronal dense core vesicle	Regulation of synaptic vesicle exocytosis	Down
Htt	Presynaptic cytosol, postsynaptic cytosol	Postsynapse to nucleus signaling pathway	Down
Itgb5	Integral component of synaptic membrane		Down
Itsn1	Presynaptic endocytic zone, postsynaptic actin cytoskeleton	Synaptic vesicle endocytosis, regulation of postsynapse organization, regulation of modification of postsynaptic actin cytoskeleton	Down
Kcnh1	Integral component of presynaptic membrane	Voltage-gated ion channel activity involved in regulation of presynaptic membrane potential, regulation of presynaptic cytosolic calcium levels, regulation of synaptic vesicle exocytosis	Down
Lama4	Synaptic cleft		Down
Lrfn2	Presynapse, postsynapse, integral component of postsynaptic density membrane	Modulation of chemical synaptic transmission, regulation of postsynapse organization	Down
Nlgn1	Integral component of postsynaptic specialization membrane	Synapse adhesion between pre- and post-synapse, regulation of presynapse organization, postsynaptic specialization assembly	Down
Ntng2	Anchored component of presynaptic active zone membrane	Regulation of presynapse assembly, synapse adhesion between pre- and post-synapse, modulation of chemical synaptic transmission, postsynaptic specialization assembly	Down
P2rx3	Integral component of presynaptic membrane	Modulation of chemical synaptic transmission	Down
P2ry1	Integral component of presynaptic active zone membrane	Regulation of presynaptic cytosolic calcium levels, regulation of synaptic vesicle exocytosis	Down
Pcdh15	Presynapse		Down
Rims2	Presynaptic active zone cytoplasmic component	Synaptic vesicle docking and priming, structural constituent of active zone, regulation of calcium-dependent activation of synaptic vesicle fusion	Down
Rims3	Postsynaptic cytosol	Regulation of synapse organization	Down
Rps10	Synapse, postsynaptic ribosome, presynaptic ribosome	Translation at presynapse, translation at postsynapse	Down
Rpsa	Synapse, postsynaptic ribosome		Down
Slc29a1	Presynapse, postsynapse		Down
Slc6a2	Integral component of presynaptic membrane, integral component of synaptic vesicle membrane	Neurotransmitter reuptake	Down
Srcin1		Regulation of synapse assembly, postsynaptic actin cytoskeleton organization	Down
Sv2c	Regulation of synaptic vesicle exocytosis	Integral component of synaptic vesicle membrane	Down
Syne1	Postsynaptic actin cytoskeleton, postsynaptic endocytic zone	Regulation of postsynaptic neurotransmitter receptor endocytosis	Down
Syngap1	Postsynaptic density, intracellular component	Modulation of chemical synaptic transmission, maintenance of postsynaptic specialization structure	Down
Syngr1	Integral component of synaptic vesicle membrane	Regulation of synaptic vesicle cycle	Down
Tanc1	Postsynaptic density, intracellular component	Regulation of postsynapse organization	Down
Wnt3a	Synapse	Modulation of chemical synaptic transmission, regulation of synapse organization, regulation of postsynapse to nucleus signaling pathway, regulation of presynapse assembly	Down

### Adult mice exposed to ethanol during development exhibit alterations in the expression patterns of genes related to mitochondria

**Fig. 5:**
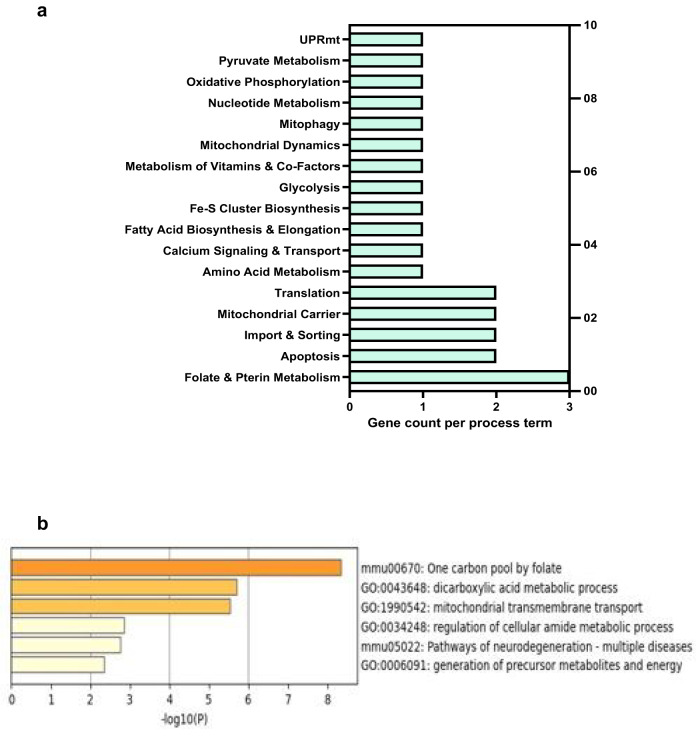
Dysregulation of mitochondria-related genes in the brains of adult mice following developmental ethanol exposure. **a** Bioinformatic analysis of 376 differentially expressed genes induced by ethanol in the brains of P60 mice to define the ethanol-induced dysregulated mitochondrial genes involved in mitochondrial functions through the mitoXplorer database. The analysis revealed 23 dysregulated mitochondrial genes with 22 genes downregulated and one gene upregulated, involved in various mitochondrial activities and metabolism. The horizontal bar graph shows the mitochondrial gene count per metabolism or mitochondrial activity. Further details on the 23 mitochondrial genes are provided in Table 2. **b** Gene ontology (GO) analysis of pathway and process of ethanol-induced dysregulated mitochondrial genes using the Metascape database. The bar graph shows the enriched terms across mitochondrial gene lists.

We utilized the mitoXplorer database [46] to analyze 376 ethanol-induced dysregulated genes (Table S1) in the brains of P60 mice. Our analysis identified 23 mitochondria-related genes that were dysregulated by ethanol, with 22 downregulated and 1 upregulated genes (Table 2). These genes were associated with 17 different mitochondrial activities, including folate and pterin metabolism, apoptosis, mitochondrial dynamics, and import and sorting of mitochondrial proteins. Interestingly, all 17 of these processes were included in the mitoXplorer database (Fig. 5a), indicating that developmental ethanol expsoure induces long-term widespread dysregulation of normal mitochondrial homeostasis. To further investigate the association of ethanol-induced dysregulated mitochondria-related genes on biological processes and functions, we conducted a GO analysis using the bioinformatic tool Metascape [47]. Our analysis revealed that ethanol exposure dysregulated genes involved in various processes, from dicarboxylic acid to amide metabolism, as well as mitochondrial transmembrane transport (Fig. 5b). While the GO enrichment data highlighted pathways related to one-carbon folate metabolism and neurodegeneration, our findings were consistent with those from mitoXplorer. Together, these unbiased in silico approaches demonstrate that ethanol exposure during development can have long-term and extensive effects on mitochondria-related gene expression in the brains of mice. The results of the Metascape analysis were consistent with the findings from mitoXplorer. This analysis provided further confirmation that the impact of ethanol on mitochondria may be extensive. However, it is noteworthy that the GO enrichment data also independently revealed pathways related to one-carbon folate metabolism and neurodegeneration.

**Table 2:** Developmental ethanol exposure-induced dysregulated mitochondrial genes in P60 mouse brains.

Gene symbol	Function annotation	Expression (ethanol vs. control)
Aldh1l2	Folate & pterin metabolism	Down
Atp5e	Oxidative phosphorylation	Down
Casp3	Apoptosis	Down
Got2	Amino acid metabolism	Down
Grpel1	Import & sorting	Down
Kdm6b	UPRmt	Down
Letm2	Calcium signaling & transport	Down
Mars2	Translation	Down
Me3	Pyruvate metabolism	Down
Mecr	Fatty acid biosynthesis & elongation	Down
Mmadhc	Metabolism of vitamins & co-factors	Down
Mthfd2l	Folate & pterin metabolism	Down
Mthfs	Folate & pterin metabolism	Down
Optn	Mitophagy	Down
Pde12	Translation	Up
Rhot2	Mitochondrial dynamics	Down
Slc25a25	Mitochondrial carrier	Down
Slc25a37	Fe-S cluster biosynthesis	Down
Slc29a1	Mitochondrial carrier	Down
Slc2a5	Glycolysis	Down
Timm17a	Import & sorting	Down
Trap1	Apoptosis	Down
Tyms	Nucleotide metabolism	Down

### Ethanol disrupts normal signaling networks associated with dysregulated synaptic and mitochondrial genes

**Fig. 6:**
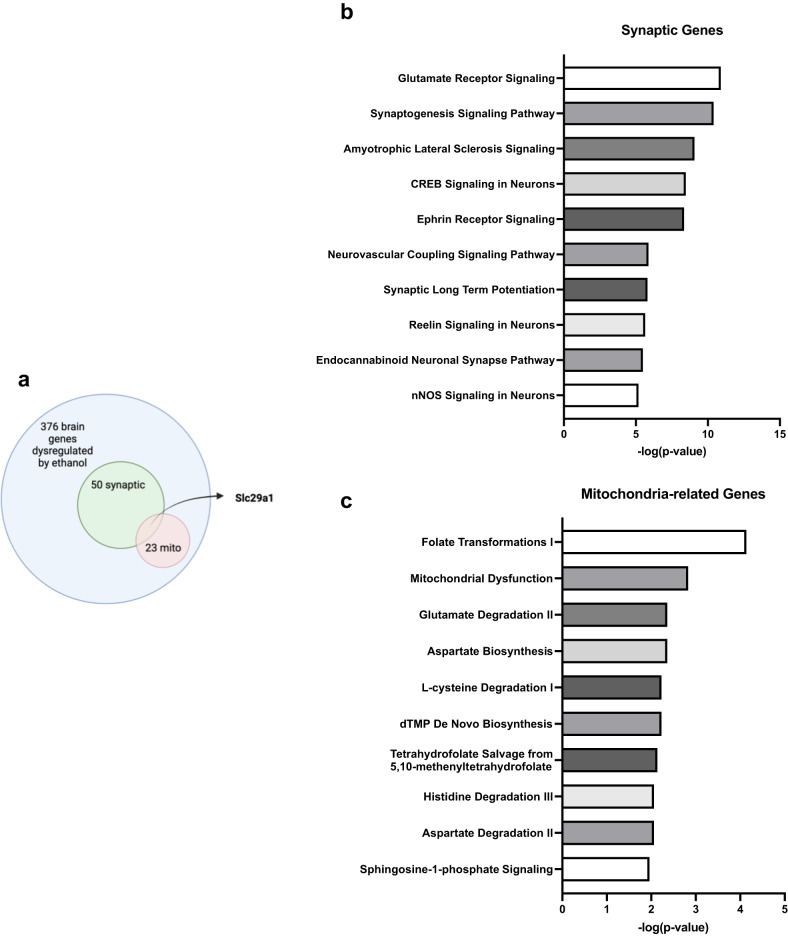
IPA analysis identified key pathways associated with dysregulation of synaptic and mitochondria-related genes following developmental ethanol exposure in P60 mouse brains. **a** Venn diagram illustrating the overlap of the differentially expressed genes used for the bioinformatics analysis. The blue circle represents the total 376 dysregulated genes, the green circle represents the 50 dysregulated synaptic genes, the red circle represents the 23 dysregulated mitochondria-related genes, and the overlapping region represents the one gene, Slc29a1, shared by both groups. **b** The top 10 canonical pathways predicted to be affected by dysregulation of synaptic genes, as determined by IPA analysis. **c** The top 10 canonical pathways predicted to be affected by dysregulation of mitochondria-related genes, as determined by IPA analysis. The complete list of genes for each pathway can be found in Table 3.

We utilized IPA bioinformatics analysis to explore the potential interaction between 50 dysregulated synaptic genes and 23 mitochondrial genes in specific canonical pathways and known diseases. Notably, among both synapse and mitochondrial datasets, the gene Slc29a1, which encodes the protein equilibrative nucleoside transporter 1 (ENT1), was the only overlapping gene (Fig. 6a). Our analysis revealed that the dysregulated synaptic genes were involved in glutamate receptor and synaptogenesis signaling pathways, as well as cAMP response element-binding protein (CREB) and Ephrin receptor signaling, among others (Fig. 6b). On the other hand, the top canonical pathways associated with the dysregulated mitochondrial genes included folate transformations, mitochondrial dysfunctions, and degradation pathways of glutamate, l-cystine, histidine, and aspartate (Fig. 6c). Table 3 listed the ethanal-induced dysregulated specific synaptic and mitochondrial genes related to each of the canonical pathways.

**Table 3:** The top 10 canonical pathways associated with developmental ethanol-induced dysregulated synapse- or mitochondria-related genes in adult mice.

	Ethanol-induced abnormally expressed genes	Canonical Pathways
Synaptic genes	Grid1, Grik1, Grik3, Grin2b, Grin2c, Grin2d, Grin3b, Grm4	Glutamate receptor signaling
	Apoe, Efna5, Efnb2, Ephb1, Grin2b, Grin2c, Grin2d, Grin3b, Grm4, Itsn1, Nlgn1, Syngap1	Synaptogenesis signaling pathway
	Cacna1c, Grid1, Grik1, Grik3, Grin2b, Grin2c, Grin2d, Grin3b	Amyotrophic lateral sclerosis signaling
	Cacna1c, Chrm1, Cnr1, Grid1, Grik1, Grik3, Grin2b, Grin2c, Grin2d, Grm4, Htr1a, Htr1d, P2ry1	CREB signaling in neurons
	Efna5, Efnb2, Ephb1, Grin2b, Grin2c, Grin2d, Grin3b, Itgb5, Itsn1	Ephrin receptor signaling
	Cacna1c, Chrm1, Grin2b, Grin2c, Grin2d, Grin3b, P2ry1	Neurovascular coupling signaling pathway
	Cacna1c, Grin2b, Grin2c, Grin2d, Grin3b, Grm4	Synaptic long-term potentiation
	Apoe, Cnr1, Grin2b, Grin2c, Grin2d, Grin3b	Reelin signaling in neurons
	Cacna1c, Cnr1, Grin2b, Grin2c, Grin2d, Grin3b	Endocannabinoid neuronal synapse pathway
	Grin2b, Grin2c, Grin2d, Grin3b	nNOS signaling in neurons
Mitochondrial genes	Mthfd2l, Mthfs	Folate transformations I
	Atp5e, Casp3, Rhot2	Mitochondrial dysfunction
	Got2	Glutamate degradation II
	Got2	Aspartate biosynthesis
	Got2	L-cysteine degradation I
	Tyms	dTMP De novo biosynthesis
	Mthfd2l	Tetrahydrofolate Salvage from 5,10-methenyltetrahydrofolate
	Mthfd2l	Histidine degradation III
	Got2	Aspartate degradation II
	Casp3, Rhot2	Sphingosine-1-phosphate signaling

**Fig. 7:**
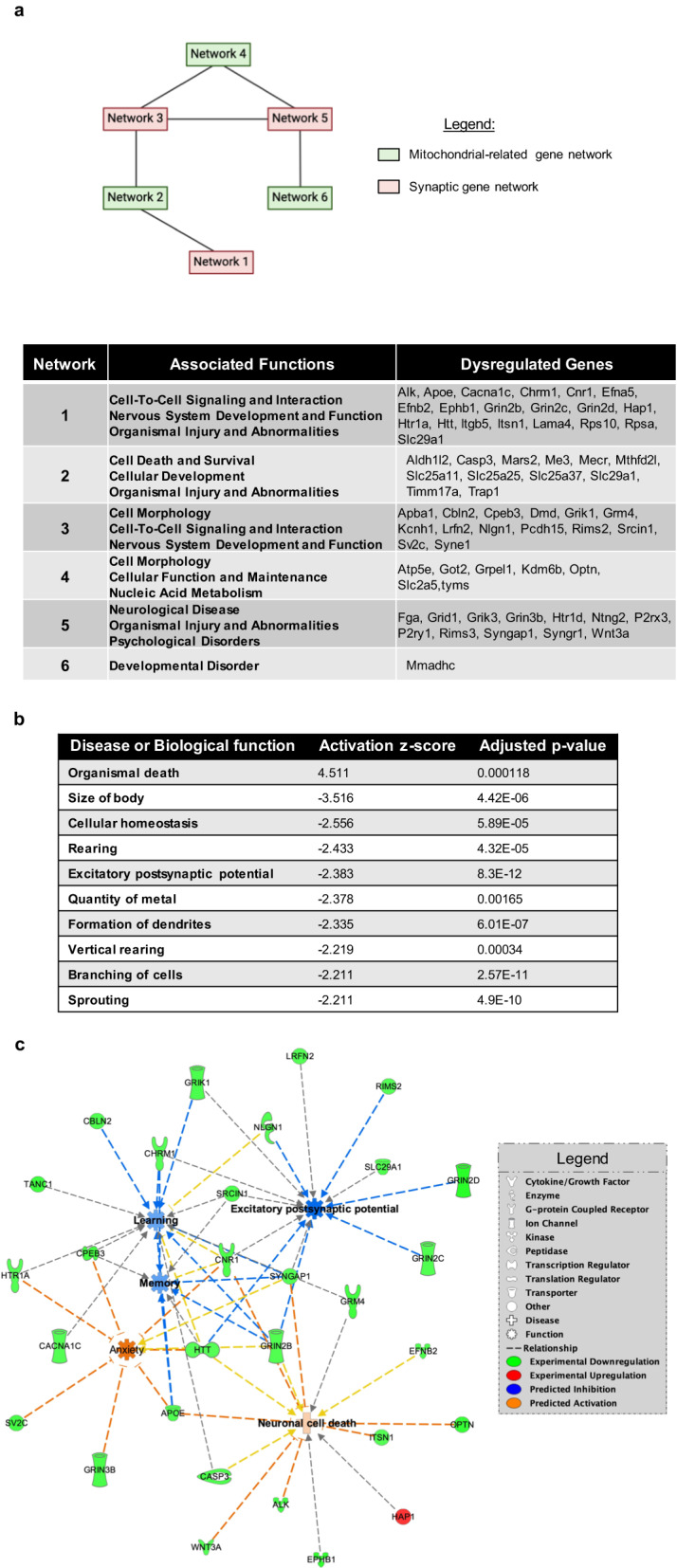
Dysregulation of synaptic and mitochondria-related genes in P60 mouse brains induced by developmental ethanol exposure were predicted to interactly influence key regulatory mechanistic networks. **a** Diagram illustrating the different connections between mechanistic networks predicted to be influenced by either synaptic or mitochondria-related gene dysregulation. In red are networks affected by ethanol-induced dysregulation of synaptic genes, and in green are those affected by dysregulation of mitochondria-related genes. The table provides details about the function of each network and the genes present in each one. **b** A table summarizing the top 10 predicted diseases and biological functions affected by the overlap between dysregulation of synaptic or mitochondria-related genes. **c** Example of the relationship between ethanol-induced gene dysregulation and predicted pathways leading to changes in learning, memory, anxiety, excitatory postsynaptic potentials, and neuronal cell death. The network highlights both synaptic or mitochondria-related genes (green genes are experimentally downregulated and red genes are upregulated), and how they are predicted to interact (activation in orange and inhibition in blue) with one or more biological functions.

Additionally, the IPA analysis of diseases and functions/networks revealed that ethanol-induced dysregulated synapse- and mitochondria-related genes are crucial in nervous system development and function, as well as various neurological diseases such as neuronal development, plasticity, cognitive dysfunction, brain damage, behavioral deficits, and neurodevelopmental disorders (Tables S2 and S3). We found significant overlaps in the affected biological networks of both synaptic and mitochondrial-related genes when comparing the IPA results from the two dysregulated datasets of genes (e.g., “Nervous System Development and Function” in networks 1 and 3; “cell morphology” in networks 3 and 4) (Fig. 7a). The findings suggest that exposure to ethanol can affect multiple networks, including dysregulated mitochondrial and synaptic gene-associated networks. These networks can potentially interact, leading to abnormal cellular development, injury, disrupted cell-to-cell signaling and interaction, and developmental disorders.

By analyzing a combined dataset of synaptic and mitochondrial-related genes, we observed the predicted activation or inhibition of various biological functions (Fig. 7b). These functions included the activation of pathways related to organismal death (z-score = 4.511), and inhibition of pathways related to excitatory postsynaptic potential (z-score = −2.383) and cellular homeostasis (z-score = −2.556). To gain a better understanding of how these dysregulated functions relate to the observed phenotypes in adult mice, we mapped the predicted pathways that lead to changes in learning, memory, anxiety, excitatory postsynaptic potentials, and neuronal cell death (Fig. 7c). The resulting network demonstrated the predicted activation of both neuronal cell death (z-score = 0.421) and anxiety (z-score = 1.882), as well as the inhibition of excitatory postsynaptic potentials (z-score = −2.383), learning (z-score = −0.844), and memory (z-score = −0.903). This finding highlights the direct relationship between synaptic and mitochondrial genes and the behavioral abnormalities induced by ethanol exposure, E/I imbalance, and gene dysregulation in mice, providing in silico confirmation of their association.

## Discussion

Prenatal exposure to ethanol can lead to long-lasting adverse effects on the brain, including changes in structure and morphology, as well as behavioral and cognitive problems. However, the cellular and molecular mechanisms underlying these effects are not well understood. This study aimed to investigate the synaptic and molecular mechanisms responsible for the long-term cognitive and behavioral effects of developmental ethanol exposure, using cellular, electrophysiological, transcriptomic, and behavioral tests. We found that neonatal mice exposed to binge-like levels of ethanol had acute neuroapoptosis and resulted in long-term anxiety-like behavior and deficits in spatial learning and memory. Additionally, there was an E/I imbalance in the hippocampal CA1 pyramidal neurons of the P60 brains of these ethanol-exposed mice. Transcriptomic analysis of adult mouse brains exposed to ethanol during development revealed dysregulation of 50 synaptic genes and 23 mitochondria-related genes. These dysregulated genes were associated with dysfunction in key biological pathways, including glutamate, CREB, and Ephrin signaling, as well as folate metabolism and the degradation of bioenergetic-related molecules. Finally, the study showed that the synaptic and mitochondria-related genes affected overlapping networks.

FASD patients commonly suffer from various long-term cognitive and mental health problems. These individuals are also 19 to 40 times more likely to become involved in the criminal justice system [54, 55]. Our findings (Fig. 2) support previous human and rodent model data showing lifelong cognitive deficits and behavioral changes. Furthermore, our study demonstrated acute widespread apoptosis in the brains and neurons of neonatal mice (Fig. 1). This developmental ethanol-induced apoptosis-induced neuronal death may contribute to long-term cognitive and mental health problems, as previously reported [56, 57]. Such apoptosis-induced cell death could act as a mechanism by itself or adversely influence the survived neurons, leading to impairment of neuronal development, synaptogenesis, synaptic plasticity, and neuronal communication, thereby causing long-term cognitive dysfunction, and behavioral problems [35, 58].

FASD currently has limited treatment options available for its long-term symptoms, prompting our recent investigation into alcohol-induced long-term cognitive and behavioral problems. Our study focused on examining long-term synaptic mechanisms and found that P60 mouse hippocampal CA1 neurons displayed an E/I imbalance, as evidenced by the decreased amplitude in sEPSCs and increases in the frequency and amplitude of sIPSCs (Fig. 3). The importance of maintaining a homeostatic E/I balance has gained attention in recent years, particularly in neurological disorders associated with impaired brain development, cognition, and abnormal behaviors [19, 59–61]. This is particularly relevant in the context of EIDN, given the dual nature of ethanol as both an agonist of GABAA receptors and an antagonist of NMDA receptors [62]. Previous studies have shown that ethanol exposure leads to deficits in NMDA receptor-specific long-term potentiation in the CA1 region of rats [63]. Additionally, organotypic immature hippocampal slices chronically exposed to ethanol showed a reduction in the frequency of sEPSCs, with an increase observed during ethanol withdrawal [64]. Our findings (Fig. 3) were consistent with these previous observations, with our study being the first to identify the specific phenotype of decreased excitation and increased inhibition in the CA1 region of mice exposed to a single binge drinking-like episode. As we discussed in the introduction, the E/I balance is crucial for maintaining normal behavior and cognition [17]. Thus, the imbalanced E/I might be an important mechanism underlying ethanol-induced cognitive dysfunctions and abnormal behaviors.

Due to the complex nature of FASD, the field has benefited from advances in transcriptomics and other unbiased molecular methods. The Weick lab has provided an excellent example of the importance of these advances [65]. Using a human pluripotent stem cell-derived neuronal model of FASD with chronic intermittent alcohol exposure and RNA-sequencing, they found robust changes in synaptic genes related to both GABAergic and glutaminergic signaling, reinforcing the hypothesis of E/I imbalance as a key mechanism in EIDN. To investigate the molecular mechanisms underlying developmental ethanol-induced long-term abnormal synaptic activities with E/I imbalance, and impaired cognition and behavioral problems, we conducted various bioinformatical analyses of developmental ethanol-induced dysregulated 376 gene profiles in P60 mouse brains generated by Kleiber et al. [34]. We identified 50 dysregulated synaptic genes that are involved in various biological processes of synapse such as synapse formation and transmission, and synaptic vesicle cycle (Fig. 4). Specifically, 8 of these 50 dysregulated synaptic genes overlap with the study mentioned above, including Alk, Dmd, Grik3, Grm4, Hap1, Ntng2, Syne1, and Tanc1 (Table 1). Alk (ALK receptor tyrosine kinase) has been extensively shown to regulate behavioral responses to ethanol in adult animal models and humans, and it plays important roles in neurodevelopment [66, 67]. Burd et al recently suggested an interaction between Alk dysregulation in ethanol-induced disorders and the risk of neuroblastoma [68]. However, no studies have yet focused on the importance of Alk in the pathophysiology of FASD Similarly, specific polymorphisms of Grik3 (glutamate ionotropic receptor kainate type subunit 3) have been associated with AUD in humans [63], but the role of this gene in FASD or EIDN is not known. Further studies can start to unravel how ethanol-induced dysregulation of the genes indicated by our work might lead to specific abnormal neuronal function and synaptic activities, such as E/I imbalance, later in life, especially considering the known increased risk of AUD in patients with FASD.

Mitochondria play a crucial role in synaptic form and function, and both animal models and human brain organoids have shown acute toxic effects of ethanol exposure on mitochondrial structure, dynamics, and function [26–28]. However, the interplay between ethanol-induced abnormal mitochondrial and synaptic signaling is not known. Our bioinformatic analysis revealed that developmental ethanol exposure resulted in 23 dysregulated mitochondrial genes in P60 mouse brains, with these genes playing important roles in mitochondrial biological processes and functions such as protein translation, metabolism, calcium signaling, and mitochondrial dynamics. Furthermore, we observed overlaps between dysregulated synaptic gene- and mitochondrial gene-related signaling networks, with some of these dysregulated genes contributing to neuronal cell death, abnormal synaptic activity, learning, memory, and anxiety. While some genes may contribute to a specific phenotype [e.g., upregulation of Hap1 (huntingtin associated protein 1) leading to detrimental effects on cell survival], others may have more overarching regulatory roles [e.g., downregulation of Grin2b (glutamate ionotropic receptor NMDA type subunit 2B) resulting in predicted inhibition of excitatory postsynaptic potentials, learning, and memory, and predicted activation of anxiety and neuronal cell death pathways]. These unbiased analyses not only validate our experimental findings but also emphasize the importance of taking a multifactorial approach when examining the phenotypes associated with EIDN.

It is worth noting that the downregulation of genes was more prevalent than upregulation, as seen in Tables 1 and 2, a finding that has been previously reported in a meta-analysis of transcriptomic datasets related to prenatal alcohol exposure [69]. This could be due to a reduction in cell population in adult mice brains caused by ethanol-induced neuroapoptosis during development [70]. Furthermore, the gene Slc29a1 is an excellent example of the novel associations our analyses found, as it overlaps both synaptic and mitochondrial processes. Slc29a1 encodes the ENT1 (equilibrative nucleoside transporter 1) protein, which is one of the primary transporters that regulate the cellular uptake of nucleosides in the brain [71]. Previous studies have shown that ethanol selectively blocks the uptake of adenosine by inhibiting ENT1, which regulates ethanol preference in adult mice by increasing the inhibitory action on nearby cells [71]. In our study, we also observed ethanol-induced long-term increased inhibitability (Fig. 3). Additionally, Slc29a1 is essential for the astrocyte-dependent metabolism of ethanol, with crucial links to AUD under the lenses of mitochondrial health and bioenergetics [72]. Although Slc29a1 is the only gene that spans both synaptic and mitochondria-related genes, it highlights the importance of this gene in both mitochondrial and synaptic activities and function in EIDN.

## Conclusion

This study supports previous findings showing that FASD patients often experience long-term cognitive and mental health problems. Specifically, our study is the first to comprehensively examine the effects of ethanol exposure on cognition and synaptic balance, as well as on complex mitochondrial and synaptic gene networks. Our findings strongly suggest that there is a close relationship between dysregulated mitochondrial and synaptic genes and that dysregulated mitochondrial and synaptic signaling plays a crucial role in the ethanol-induced long-term abnormal synaptic activities with E/I imbalance and impairment of cognition and behavior in adult mice. Moreover, our results indicate that the widespread symptoms surrounding FASD are unlikely caused by a single dysregulated gene or family of genes alone. Instead, the compounded dysregulation of synaptic and mitochondria-related genes, in the context of persistent behavioral, cognitive, and electrophysiological abnormalities, represents an unprecedented step towards understanding the full spectrum of the pathologies caused by prenatal alcohol exposure. Our findings also lay the foundation for further studies examining the effects of ethanol exposure on synaptic function and mitochondrial health under different dosages, frequencies, and developmental stages. By providing insights into the molecular, mitochondrial bioenergetic, synaptic activity, cognitive, and behavioral changes caused by ethanol exposure, this study sheds light on the cellular and molecular mechanisms underlying the long-term cognitive and behavioral effects of developmental ethanol exposure. Finally, our integrative approaches offer the potential for the development of specific and effective prevention and intervention strategies against FASD-associated long-term cognitive and mental problems by targeting specific molecules, signaling, and networks.

### Supplementary information

Supplementary Table Legend

Table S1

Table S2

Table S3

## Data Availability

All data generated or analyzed during this study are included in this published article and its supplementary information files.
