# Supplementary material for: Synaptic and mitochondrial mechanisms behind alcohol-induced imbalance of excitatory/inhibitory synaptic activity and associated cognitive and behavioral abnormalities
Source: Transl Psychiatry. 2024 Jan 22;14:51. doi: 10.1038/s41398-024-02748-8 (PMC10803756; doi:10.1038/s41398-024-02748-8)
Supplement: Supplementary file 1 — Supplementary Table Legend [file 41398_2024_2748_MOESM1_ESM.docx]

**Supplementary information**

Supplemental material includes Table S1 to 3.

**Table S1**. Developmental ethanol exposure-induced dysregulated genes in P60 mouse brains

**Table S2**. The developmental ethanol exposure-induced dysregulated synaptic genes in P60 mouse brains that are associated with psychological disorders and cognitive dysfunction.

**Table S3**. The association of P7 ethanol exposure-induced dysregulated mitochondrial metabolism-related genes in P60 mouse brains with neurological diseases.
