# Supplementary material for: Synaptic and mitochondrial mechanisms behind alcohol-induced imbalance of excitatory/inhibitory synaptic activity and associated cognitive and behavioral abnormalities
Source: Transl Psychiatry. 2024 Jan 22;14:51. doi: 10.1038/s41398-024-02748-8 (PMC10803756; doi:10.1038/s41398-024-02748-8)
Supplement: Supplementary file 2 — Table S1 [file 41398_2024_2748_MOESM2_ESM.docx]

| **Table S1. Developmental ethanol exposure-induced dysregulated genes in P60 mouse brains [34]** | | | | | |
| --- | --- | --- | --- | --- | --- |
| **Gene assignment** | **Gene Symbol** | **RefSeq** | **p-value** | **Fold-change** |  |
| NM_019816 // Aatf // apoptosis antagonizing transcription factor // 11\|11 B5 // 56321 / | Aatf | NM_019816 | 0.000562 | -1.31696 |  |
| NM_175314 // Adamts9 // a disintegrin-like and metallopeptidase (reprolysin type) with | Adamts9 | NM_175314 | 1.67E-05 | -1.22885 |  |
| NM_007409 // Adh1 // alcohol dehydrogenase 1 (class I) // 3 G3\|3 71.2 cM // 11522 /// E | Adh1 | NM_007409 | 5.26E-05 | -1.34906 |  |
| NM_177078 // Adrbk2 // adrenergic receptor kinase, beta 2 // 5 F\|5 60.0 cM // 320129 // (G protein-coupled receptor kinase 3) | Adrbk2 (GRK3) | NM_177078 | 0.000485 | -1.27646 |  |
| NM_026531 // Aen // apoptosis enhancing nuclease // 7\|7 D2 // 68048 /// NM_001162939 // | Aen | NM_026531 | 1.73E-05 | -1.34673 |  |
| NM_153543 // Aldh1l2 // aldehyde dehydrogenase 1 family, member L2 // 10 C1\|10 // 21618 | Aldh1l2 | NM_153543 | 4.48E-05 | -1.32406 |  |
| NM_007439 // Alk // anaplastic lymphoma kinase // 17 E1.3\|17 50.0 cM // 11682 /// ENSMU | Alk | NM_007439 | 0.000319 | -1.37218 |  |
| NM_007585 // Anxa2 // annexin A2 // 9 C\|9 37.0 cM // 12306 /// ENSMUST00000034756 // An | Anxa2 | NM_007585 | 3.48E-05 | -1.42123 |  |
| NM_007455 // Ap1g2 // adaptor protein complex AP-1, gamma 2 subunit // 14 C3\|14 // 1176 | Ap1g2 | NM_007455 | 0.000158 | -1.36451 |  |
| NM_177034 // Apba1 // amyloid beta (A4) precursor protein binding, family A, member 1 / | Apba1 | NM_177034 | 9.30E-05 | -1.24777 |  |
| NM_009696 // Apoe // apolipoprotein E // 7 A3\|7 4.0 cM // 11816 /// ENSMUST00000003066 | Apoe | NM_009696 | 1.63E-05 | -1.26396 |  |
| NM_177820 // Apol10b // apolipoprotein L 10b // 15 E1\|15 // 328561 /// NM_001143686 // | Apol10b | NM_177820 | 1.71E-06 | -1.26847 |  |
| NM_025983 // Atp5e // ATP synthase, H+ transporting, mitochondrial F1 complex, epsilon | Atp5e | NM_025983 | 2.61E-05 | -1.34942 |  |
| NM_019864 // Atr // ataxia telangiectasia and Rad3 related // 9 E4\|9 // 245000 /// ENSM | Atr | NM_019864 | 1.45E-05 | -1.36915 |  |
| NM_029705 // Atxn3 // ataxin 3 // 12 E\|12 // 110616 /// NM_001167914 // Atxn3 // ataxin | Atxn3 | NM_029705 | 0.000795 | -1.34194 |  |
| NM_130452 // Bbox1 // butyrobetaine (gamma), 2-oxoglutarate dioxygenase 1 (gamma-butyro | Bbox1 | NM_130452 | 1.77E-05 | -1.4246 |  |
| NM_025840 // Bzw2 // basic leucine zipper and W2 domains 2 // 12\|12 B2 // 66912 /// ENS | Bzw2 | NM_025840 | 4.38E-07 | -1.24262 |  |
| NM_001159533 // Cacna1c // calcium channel, voltage-dependent, L type, alpha 1C subunit | Cacna1c | NM_001159533 | 5.36E-06 | -1.24499 |  |
| NM_028500 // Calr3 // calreticulin 3 // 8\|8 C1 // 73316 /// NM_029782 // Calr3 // calre | Calr3 | NM_028500 | 2.46E-05 | -1.27475 |  |
| NM_007601 // Capn3 // calpain 3 // 2 E5\|2 67.2 cM // 12335 /// NM_001177799 // Capn3 // | Capn3 | NM_007601 | 0.000147 | -1.24624 |  |
| NM_009810 // Casp3 // caspase 3 // 8 B1.1\|8 26.0 cM // 12367 /// ENSMUST00000093517 // | Casp3 | NM_009810 | 0.000764 | -1.31333 |  |
| NM_172633 // Cbln2 // cerebellin 2 precursor protein // 18 E4\|18 54.0 cM // 12405 /// E | Cbln2 | NM_172633 | 0.000705 | -1.27091 |  |
| NM_011800 // Cdh20 // cadherin 20 // 1 E2.1\|1 59.0 cM // 23836 /// ENSMUST00000062528 / | Cdh20 | NM_011800 | 0.000434 | -1.31014 |  |
| NM_145990 // Cdk5rap2 // CDK5 regulatory subunit associated protein 2 // 4 C2\|4 // 2144 | Cdk5rap2 | NM_145990 | 0.000346 | -1.26238 |  |
| NM_177647 // Cdnf // cerebral dopamine neurotrophic factor // 2 A1\|2 // 227526 /// ENSM | Cdnf | NM_177647 | 9.65E-06 | -1.54899 |  |
| NM_198019 // Cep78 // centrosomal protein 78 // 19 A\|19 // 208518 /// ENSMUST0000004770 | Cep78 | NM_198019 | 0.000227 | -1.37292 |  |
| NM_207298 // Cercam // cerebral endothelial cell adhesion molecule // 2 B\|2 19.0 cM // | Cercam | NM_207298 | 0.000777 | -1.40995 |  |
| NM_026885 // Chmp2a // chromatin modifying protein 2A // 7 A2\|7 // 68953 /// ENSMUST000 | Chmp2a | NM_026885 | 1.50E-05 | -1.27191 |  |
| NM_001112697 // Chrm1 // cholinergic receptor, muscarinic 1, CNS // 19 A\|19 // 12669 // | Chrm1 | NM_001112697 | 0.000361 | -1.23802 |  |
| NM_027562 // Clec2g // C-type lectin domain family 2, member g // 6 F3\|6 // 70809 /// N | Clec2g | NM_027562 | 6.56E-06 | -1.24427 |  |
| NM_053155 // Clmn // calmin // 12\|12 F1 // 94040 /// NM_001040682 // Clmn // calmin // | Clmn | NM_053155 | 1.35E-05 | -1.48616 |  |
| NM_001033242 // Cln5 // ceroid-lipofuscinosis, neuronal 5 // 14 E2.3\|14 53.56 cM // 211 | Cln5 | NM_001033242 | 0.000677 | -1.36471 |  |
| NM_133840 // Clp1 // CLP1, cleavage and polyadenylation factor I subunit, homolog (S. c | Clp1 | NM_133840 | 0.000147 | -1.20448 |  |
| NM_023493 // Cml5 // camello-like 5 // 6 C3\|6 // 69049 /// ENSMUST00000032074 // Cml5 / | Cml5 | NM_023493 | 0.000618 | -1.46947 |  |
| NM_027022 // Cmtm2a // CKLF-like MARVEL transmembrane domain containing 2A // 8 D1\|8 // | Cmtm2a | NM_027022 | 1.16E-05 | -1.35867 |  |
| NM_007726 // Cnr1 // cannabinoid receptor 1 (brain) // 4 A5\|4 13.9 cM // 12801 /// ENSM | Cnr1 | NM_007726 | 0.000344 | -1.29841 |  |
| NM_198300 // Cpeb3 // cytoplasmic polyadenylation element binding protein 3 // 19 C2\|19 | Cpeb3 | NM_198300 | 4.63E-05 | -1.3689 |  |
| ENSMUST00000100216 // Crebbp // CREB binding protein // 16 A1 // 12914 | Crebbp | ENSMUST00000100216 | 0.0005 | -1.2534 |  |
| NM_001160265 // Cyp2w1 // cytochrome P450, family 2, subfamily w, polypeptide 1 // 5 G2 | Cyp2w1 | NM_001160265 | 2.14E-05 | -1.27472 |  |
| NM_001177964 // Dcdc2c // doublecortin domain containing 2C // 12 A2\|12 // 68511 /// EN | Dcdc2c | NM_001177964 | 1.69E-07 | -1.37829 |  |
| NM_207677 // Dedd2 // death effector domain-containing DNA binding protein 2 // 7 A3\|7 | Dedd2 | NM_207677 | 0.000199 | -1.39171 |  |
| NM_139219 // Defb9 // defensin beta 9 // 8 A2\|8 // 246079 /// ENSMUST00000057076 // Def | Defb9 | NM_139219 | 2.44E-05 | -1.40784 |  |
| NM_022408 // Dgcr14 // DiGeorge syndrome critical region gene 14 // 16 B1-B3\|16 10.45 c | Dgcr14 | NM_022408 | 4.99E-06 | -1.24447 |  |
| NM_023178 // Dmap1 // DNA methyltransferase 1-associated protein 1 // 4\|4 D1 // 66233 / | Dmap1 | NM_023178 | 0.000993 | -1.37991 |  |
| NM_007868 // Dmd // dystrophin, muscular dystrophy // X C\|X 32.0 cM // 13405 /// ENSMUS | Dmd | NM_007868 | 4.68E-05 | -1.30015 |  |
| NM_001163026 // Dnajc13 // DnaJ (Hsp40) homolog, subfamily C, member 13 // 9 F1\|9 // 23 | Dnajc13 | NM_001163026 | 0.000703 | -1.21414 |  |
| NM_008929 // Dnajc3 // DnaJ (Hsp40) homolog, subfamily C, member 3 // 14 E4\|14 // 10003 | Dnajc3 | NM_008929 | 8.09E-05 | -1.37975 |  |
| NM_134448 // Dst // dystonin // 1 B\|1 16.5 cM // 13518 /// NM_133833 // Dst // dystonin | Dst | NM_134448 | 6.64E-06 | -1.24065 |  |
| NM_145549 // Efcab7 // EF-hand calcium binding domain 7 // 4 C6\|4 // 230500 /// ENSMUST | Efcab7 | NM_145549 | 1.75E-05 | -1.42687 |  |
| NM_207654 // Efna5 // ephrin A5 // 17 E1.1\|17 33.5 cM // 13640 /// NM_010109 // Efna5 / | Efna5 | NM_207654 | 0.000349 | -1.32965 |  |
| NM_010111 // Efnb2 // ephrin B2 // 8 A1.1\|8 2.0 cM // 13642 /// ENSMUST00000001319 // E | Efnb2 | NM_010111 | 0.000319 | -1.29568 |  |
| NM_207655 // Egfr // epidermal growth factor receptor // 11 A1-A4\|11 9.0 cM // 13649 // | Egfr | NM_207655 | 2.50E-05 | -1.26519 |  |
| NM_028133 // Egln3 // EGL nine homolog 3 (C. elegans) // 12 C1\|12 // 112407 /// ENSMUST | Egln3 | NM_028133 | 0.001027 | 1.41476 |  |
| NM_007923 // Elk4 // ELK4, member of ETS oncogene family // 1\|1 E3-G // 13714 /// ENSMU | Elk4 | NM_007923 | 0.000226 | -1.31379 |  |
| AK082061 // Ephb1 // Eph receptor B1 // 9 F1\|9 // 270190 | Ephb1 | AK082061 | 0.000278 | -1.49412 |  |
| NM_027984 // Epn3 // epsin 3 // 11\|11 C // 71889 /// ENSMUST00000127305 // Epn3 // epsi | Epn3 | NM_027984 | 6.49E-09 | -1.40649 |  |
| NM_007959 // Etv2 // ets variant gene 2 // 7 B1\|7 // 14008 /// ENSMUST00000108147 // Et | Etv2 | NM_007959 | 1.94E-06 | -1.23242 |  |
| NM_010175 // Fadd // Fas (TNFRSF6)-associated via death domain // 7 F5\|7 70.0 cM // 140 | Fadd | NM_010175 | 0.000591 | -1.24393 |  |
| NM_019833 // Fam69b // family with sequence similarity 69, member B // 2 A3\|2 // 56279 | Fam69b | NM_019833 | 2.61E-05 | -1.27964 |  |
| NM_007990 // Fau // Finkel-Biskis-Reilly murine sarcoma virus (FBR-MuSV) ubiquitously e | Fau | NM_007990 | 0.000993 | -1.24173 |  |
| NM_007992 // Fbln2 // fibulin 2 // 6 D\|6 37.2 cM // 14115 /// NM_001081437 // Fbln2 // | Fbln2 | NM_007992 | 1.20E-06 | -1.32592 |  |
| NM_172748 // Fbxl19 // F-box and leucine-rich repeat protein 19 // 7 F3\|7 // 233902 /// | Fbxl19 | NM_172748 | 5.00E-06 | -1.37952 |  |
| NM_001111048 // Fga // fibrinogen alpha chain // 3 F1\|3 44.8 cM // 14161 /// NM_010196 | Fga | NM_001111048 | 5.28E-05 | -1.23387 |  |
| NM_013518 // Fgf9 // fibroblast growth factor 9 // 14 D\|14 21.0 cM // 14180 /// ENSMUST | Fgf9 | NM_013518 | 7.39E-05 | -1.30274 |  |
| NM_178887 // Fibcd1 // fibrinogen C domain containing 1 // 2 B\|2 // 98970 /// BC060634 | Fibcd1 | NM_178887 | 7.12E-06 | -1.28068 |  |
| NM_010216 // Figf // c-fos induced growth factor // X F5\|X 70.0 cM // 14205 /// ENSMUST | Figf | NM_010216 | 0.00058 | -1.2314 |  |
| NM_016770 // Folh1 // folate hydrolase // 7\|7 D1-D2 // 53320 /// NM_001159706 // Folh1 | Folh1 | NM_016770 | 0.000152 | -1.27501 |  |
| NM_010426 // Foxf1a // forkhead box F1a // 8 E1\|8 67.0 cM // 15227 /// ENSMUST000000506 | Foxf1a | NM_010426 | 2.34E-05 | -1.35527 |  |
| NM_001081172 // Frmpd1 // FERM and PDZ domain containing 1 // 4 B1\|4 // 666060 /// ENSM | Frmpd1 | NM_001081172 | 5.19E-06 | -1.65472 |  |
| NM_001113478 // Frrs1 // ferric-chelate reductase 1 // 3 G1\|3 // 20321 /// NM_009146 // | Frrs1 | NM_001113478 | 2.90E-05 | -1.24241 |  |
| NM_144939 // Frs3 // fibroblast growth factor receptor substrate 3 // 17 C\|17 // 107971 | Frs3 | NM_144939 | 2.36E-05 | -1.40849 |  |
| NM_028194 // Fryl // furry homolog-like (Drosophila) // 5 C3.2\|5 // 72313 /// ENSMUST00 | Fryl | NM_028194 | 0.000419 | -1.28428 |  |
| NM_010240 // Ftl1 // ferritin light chain 1 // 7 B4\|7 // 14325 /// NM_008049 // Ftl2 // | Ftl1 | NM_010240 | 7.07E-05 | -1.51031 |  |
| NM_172283 // Fuk // fucokinase // 8 E1\|8 // 234730 /// NM_181666 // Fuk // fucokinase / | Fuk | NM_172283 | 0.000595 | -1.48885 |  |
| NM_134161 // Fut10 // fucosyltransferase 10 // 8 A3\|8 // 171167 /// NM_001012517 // Fut | Fut10 | NM_134161 | 1.46E-05 | -1.58376 |  |
| NM_013716 // G3bp1 // Ras-GTPase-activating protein SH3-domain binding protein 1 // 11 | G3bp1 | NM_013716 | 0.0005 | -1.32566 |  |
| ENSMUST00000034428 // Gabarapl2 // gamma-aminobutyric acid (GABA) A receptor-associated | Gabarapl2 | ENSMUST00000034428 | 0.042977 | -1.51155 |  |
| NM_001081190 // Gabrr3 // gamma-aminobutyric acid (GABA) receptor, rho 3 // 16 C1.3\|16 | Gabrr3 | NM_001081190 | 0.012305 | -1.20698 |  |
| NM_175154 // Galk2 // galactokinase 2 // 2 F1\|2 // 69976 /// ENSMUST00000028636 // Galk | Galk2 | NM_175154 | 0.000213 | -1.31732 |  |
| NM_175032 // Galntl6 // UDP-N-acetyl-alpha-D-galactosamine:polypeptide N-acetylgalactos | Galntl6 | NM_175032 | 0.000571 | 1.29377 |  |
| NM_008095 // Gbas // glioblastoma amplified sequence // 5 G1.3\|5 // 14467 /// ENSMUST00 | Gbas | NM_008095 | 0.000722 | -1.32302 |  |
| NM_001166065 // Gcnt4 // glucosaminyl (N-acetyl) transferase 4, core 2 (beta-1,6-N-acet | Gcnt4 | NM_001166065 | 0.000825 | -1.44369 |  |
| NM_013529 // Gfpt2 // glutamine fructose-6-phosphate transaminase 2 // 11 B1.2\|11 26.0 | Gfpt2 | NM_013529 | 3.41E-05 | -1.25641 |  |
| NM_031247 // Gimap3 // GTPase, IMAP family member 3 // 6 B2.3\|6 // 83408 /// ENSMUST000 | Gimap3 | NM_031247 | 0.000537 | -1.22822 |  |
| NM_028608 // Glipr1 // GLI pathogenesis-related 1 (glioma) // 10\|10 D1 // 73690 /// NM_ | Glipr1 | NM_028608 | 0.000309 | -1.25669 |  |
| NM_023140 // Glrx3 // glutaredoxin 3 // 7\|7 F5 // 30926 /// ENSMUST00000064404 // Glrx3 | Glrx3 | NM_023140 | 0.000359 | -1.87237 |  |
| NM_001081418 // Gltscr1 // glioma tumor suppressor candidate region gene 1 // 7 A2\|7 15 | Gltscr1 | NM_001081418 | 0.000147 | -1.3041 |  |
| NM_133831 // Gltscr2 // glioma tumor suppressor candidate region gene 2 // 7 A2\|7 15.13 | Gltscr2 | NM_133831 | 0.000808 | -1.3044 |  |
| NM_198169 // Gmeb2 // glucocorticoid modulatory element binding protein 2 // 2 H4\|2 // | Gmeb2 | NM_198169 | 0.000222 | -1.30686 |  |
| NM_013530 // Gnb3 // guanine nucleotide binding protein (G protein), beta 3 // 6 F2\|6 6 | Gnb3 | NM_013530 | 4.21E-07 | -1.41459 |  |
| NM_011937 // Gnpda1 // glucosamine-6-phosphate deaminase 1 // 18 B3\|18 // 26384 /// NR_ | Gnpda1 | NM_011937 | 8.42E-05 | -1.47628 |  |
| NM_019425 // Gnpnat1 // glucosamine-phosphate N-acetyltransferase 1 // 14 C1\|14 // 5434 | Gnpnat1 | NM_019425 | 0.000336 | -1.25443 |  |
| NM_010325 // Got2 // glutamate oxaloacetate transaminase 2, mitochondrial // 8 D1\|8 46. | Got2 | NM_010325 | 0.000462 | -1.33541 |  |
| NM_173747 // Gpkow // G patch domain and KOW motifs // X A1.1\|X 1.6 cM // 209416 /// EN | Gpkow | NM_173747 | 5.44E-06 | 1.34875 |  |
| NM_176912 // Gpr77 // G protein-coupled receptor 77 // 7 A2\|7 // 319430 /// NM_00114600 | Gpr77 | NM_176912 | 0.00103 | -1.28356 |  |
| NM_008166 // Grid1 // glutamate receptor, ionotropic, delta 1 // 14 B\|14 13.5 cM // 148 | Grid1 | NM_008166 | 0.000556 | -1.31137 |  |
| NM_146072 // Grik1 // glutamate receptor, ionotropic, kainate 1 // 16 C3.3\|16 58.0 cM / | Grik1 | NM_146072 | 0.001496 | -1.29636 |  |
| NM_001081097 // Grik3 // glutamate receptor, ionotropic, kainate 3 // 4 D2.2\|4 57.2 cM | Grik3 | NM_001081097 | 0.002155 | -1.29478 |  |
| NM_008171 // Grin2b // glutamate receptor, ionotropic, NMDA2B (epsilon 2) // 6 G1\|6 64. | Grin2b | NM_008171 | 0.000172 | -1.26589 |  |
| NM_010350 // Grin2c // glutamate receptor, ionotropic, NMDA2C (epsilon 3) // 11 E2\|11 7 | Grin2c | NM_010350 | 0.002277 | -1.2584 |  |
| NM_008172 // Grin2d // glutamate receptor, ionotropic, NMDA2D (epsilon 4) // 7 B4\|7 23. | Grin2d | NM_008172 | 9.16E-07 | -1.53931 |  |
| NM_130455 // Grin3b // glutamate receptor, ionotropic, NMDA3B // 10 C1\|10 // 170483 /// | Grin3b | NM_130455 | 0.000635 | -1.30702 |  |
| NM_001013385 // Grm4 // glutamate receptor, metabotropic 4 // 17 A3.3\|17 // 268934 /// | Grm4 | NM_001013385 | 0.003564 | -1.38447 |  |
| NM_024478 // Grpel1 // GrpE-like 1, mitochondrial // 5 B3\|5 // 17713 /// ENSMUST0000003 | Grpel1 | NM_024478 | 0.000125 | -1.38377 |  |
| NM_008178 // Gsx1 // GS homeobox 1 // 5 G3\|5 41.0 cM // 14842 /// ENSMUST00000065382 // | Gsx1 | NM_008178 | 1.29E-05 | -1.36706 |  |
| NM_010373 // Gzme // granzyme E // 14 C3\|14 20.5 cM // 14942 /// ENSMUST00000089549 // | Gzme | NM_010373 | 0.027337 | -1.20631 |  |
| NM_010404 // Hap1 // huntingtin-associated protein 1 // 11 D\|11 60.0 cM // 15114 /// NM | Hap1 | NM_010404 | 3.60E-05 | 1.50377 |  |
| NM_173400 // Haus6 // HAUS augmin-like complex, subunit 6 // 4 C4\|4 42.6 cM // 230376 / | Haus6 | NM_173400 | 7.68E-05 | -1.42012 |  |
| NM_001081192 // Hcn4 // hyperpolarization-activated, cyclic nucleotide-gated K+ 4 // 9\| | Hcn4 | NM_001081192 | 0.000353 | -1.52613 |  |
| NM_199198 // Hdac10 // histone deacetylase 10 // 15 E3\|15 // 170787 /// NR_028447 // Hd | Hdac10 | NM_199198 | 0.000909 | -1.25187 |  |
| NM_027382 // Hdac8 // histone deacetylase 8 // X\|X C3 // 70315 /// ENSMUST00000087916 / | Hdac8 | NM_027382 | 0.000455 | -1.38268 |  |
| NM_144835 // Heatr1 // HEAT repeat containing 1 // 13 A1\|13 8.0 cM // 217995 /// ENSMUS | Heatr1 | NM_144835 | 0.000537 | -1.32609 |  |
| NM_175244 // Hectd3 // HECT domain containing 3 // 4 D1\|4 // 76608 /// ENSMUST000000500 | Hectd3 | NM_175244 | 5.18E-05 | -1.31528 |  |
| NM_175256 // Heg1 // HEG homolog 1 (zebrafish) // 16 B3\|16 // 77446 /// ENSMUST00000038 | Heg1 | NM_175256 | 0.002295 | -1.23289 |  |
| NM_175256 // Heg1 // HEG homolog 1 (zebrafish) // 16 B3\|16 // 77446 | Heg1 | NM_175256 | 0.000257 | -1.54582 |  |
| NM_198298 // Helz // helicase with zinc finger domain // 11 E1\|11 // 78455 /// ENSMUST0 | Helz | NM_198298 | 6.71E-06 | -1.25906 |  |
| NM_178899 // Hepacam2 // HEPACAM family member 2 // 6 A1\|6 // 101202 /// ENSMUST0000004 | Hepacam2 | NM_178899 | 4.75E-06 | -1.31981 |  |
| NM_010421 // Hexa // hexosaminidase A // 9 B\|9 29.0 cM // 15211 /// ENSMUST00000026262 | Hexa | NM_010421 | 1.93E-05 | -1.38771 |  |
| NM_178195 // Hist1h2bf // histone cluster 1, H2bf // 13 A2-A3\|13 // 319180 /// NM_02342 | Hist1h2bf | NM_178195 | 2.74E-05 | -1.32736 |  |
| NM_178200 // Hist1h2bm // histone cluster 1, H2bm // 13 A2-A3\|13 // 319186 /// ENSMUST0 | Hist1h2bm | NM_178200 | 0.000866 | -1.53468 |  |
| [NM_175653 // Hist1h3c // histone cluster 1, H3c // 13 A2-A3\|13 // 319148 /// NM_178203](http://www.ncbi.nlm.nih.gov/entrez/query.fcgi?db=Gene&cmd=search&term=Hist1h3c) | Hist1h3c | NM_175653 | 0.000939 | -1.30067 |  |
| NM_175653 // Hist1h3c // histone cluster 1, H3c // 13 A2-A3\|13 // 319148 /// NM_178203 | Hist1h3c | NM_175653 | 0.000939 | -1.30067 |  |
| NM_175654 // Hist1h4d // histone cluster 1, H4d // 13 A2-A3\|13 // 319156 | Hist1h4d | NM_175654 | 2.72E-05 | -1.26488 |  |
| NM_033596 // Hist2h4 // histone cluster 2, H4 // 3\|3 F1-F2 // 97122 /// BC057955 // His | Hist2h4 | NM_033596 | 8.79E-06 | -1.26878 |  |
| NM_009210 // Hltf // helicase-like transcription factor // 3 A2\|3 12.0 cM // 20585 /// | Hltf | NM_009210 | 1.86E-06 | -1.33408 |  |
| NM_001024720 // Hmcn1 // hemicentin 1 // 1 G1\|1 // 545370 /// ENSMUST00000074783 // Hmc | Hmcn1 | NM_001024720 | 0.001001 | -1.27612 |  |
| NM_008296 // Hsf1 // heat shock factor 1 // 15 D3\|15 43.0 cM // 15499 /// ENSMUST000000 | Hsf1 | NM_008296 | 0.013201 | -1.21859 |  |
| NM_008308 // Htr1a // 5-hydroxytryptamine (serotonin) receptor 1A // 13 D1\|13 58.0 cM / | Htr1a | NM_008308 | 0.000564 | -1.24518 |  |
| NM_008309 // Htr1d // 5-hydroxytryptamine (serotonin) receptor 1D // 4 D3\|4 66.0 cM // | Htr1d | NM_008309 | 0.009049 | -1.27773 |  |
| NM_010483 // Htr5b // 5-hydroxytryptamine (serotonin) receptor 5B // 1 E4-G\|1 63.0 cM / | Htr5b | NM_010483 | 0.011939 | 1.30343 |  |
| NM_010414 // Htt // huntingtin // 5 B2\|5 20.0 cM // 15194 /// ENSMUST00000080036 // Htt | Htt | NM_010414 | 0.001082 | -1.22779 |  |
| NM_030694 // Ifitm2 // interferon induced transmembrane protein 2 // 7\|7 F5 // 80876 // | Ifitm2 | NM_030694 | 0.000497 | -1.4336 |  |
| NM_025903 // Ifrd2 // interferon-related developmental regulator 2 // 9 F1\|9 60.21 cM / | Ifrd2 | NM_025903 | 8.49E-05 | -1.21031 |  |
| NM_009951 // Igf2bp1 // insulin-like growth factor 2 mRNA binding protein 1 // 11 D\|11 | Igf2bp1 | NM_009951 | 1.72E-06 | -1.55567 |  |
| NM_145826 // Il17re // interleukin 17 receptor E // 6 E3\|6 // 57890 /// NM_001034031 // | Il17re | NM_145826 | 1.59E-07 | -1.27707 |  |
| NM_029646 // Il34 // interleukin 34 // 8 E1\|8 // 76527 /// NM_001135100 // Il34 // inte | Il34 | NM_029646 | 2.59E-05 | -1.4387 |  |
| NM_054079 // Iltifb // interleukin 10-related T cell-derived inducible factor beta // 1 | Iltifb | NM_054079 | 8.31E-07 | -2.19852 |  |
| NM_010567 // Inppl1 // inositol polyphosphate phosphatase-like 1 // 7 F1\|7 // 16332 /// | Inppl1 | NM_010567 | 7.16E-06 | -1.26822 |  |
| NM_010568 // Insr // insulin receptor // 8 A1.1\|8 1.0 cM // 16337 /// ENSMUST0000009129 | Insr | NM_010568 | 2.48E-05 | -1.30097 |  |
| NM_028833 // Iqce // IQ motif containing E // 5\|5 G1 // 74239 /// ENSMUST00000041783 // | Iqce | NM_028833 | 4.91E-06 | -1.34344 |  |
| NM_008390 // Irf1 // interferon regulatory factor 1 // 11 B1.3\|11 29.0 cM // 16362 /// | Irf1 | NM_008390 | 0.000523 | -1.25026 |  |
| NM_012057 // Irf5 // interferon regulatory factor 5 // 6 A3.3\|6 7.1 cM // 27056 /// ENS | Irf5 | NM_012057 | 0.00033 | -1.24549 |  |
| NM_018826 // Irx5 // Iroquois related homeobox 5 (Drosophila) // 8 C5\|8 43.3 cM // 5435 | Irx5 | NM_018826 | 0.000156 | -1.53612 |  |
| NM_020583 // Isg20 // interferon-stimulated protein // 7 D3\|7 // 57444 /// NM_001113527 | Isg20 | NM_020583 | 0.000896 | -1.51209 |  |
| NM_001082960 // Itgam // integrin alpha M // 7\|7 F4 // 16409 /// NM_008401 // Itgam // | Itgam | NM_001082960 | 0.000704 | -1.28798 |  |
| NM_001145884 // Itgb5 // integrin beta 5 // 16 B3\|16 // 16419 /// NM_010580 // Itgb5 // | Itgb5 | NM_001145884 | 0.000596 | 1.36513 |  |
| NM_025922 // Itpa // inosine triphosphatase (nucleoside triphosphate pyrophosphatase) / | Itpa | NM_025922 | 0.000726 | -1.25389 |  |
| NM_010587 // Itsn1 // intersectin 1 (SH3 domain protein 1A) // 16 C3.3-C4\|16 // 16443 / | Itsn1 | NM_010587 | 7.13E-05 | -1.23282 |  |
| NM_010600 // Kcnh1 // potassium voltage-gated channel, subfamily H (eag-related), membe | Kcnh1 | NM_010600 | 4.36E-07 | -1.26571 |  |
| NM_029911 // Kcnk10 // potassium channel, subfamily K, member 10 // 12 E\|12 // 72258 // | Kcnk10 | NM_029911 | 0.000448 | -1.31587 |  |
| NM_145997 // Kdm5a // lysine (K)-specific demethylase 5A // 6 F1\|6 54.0 cM // 214899 // | Kdm5a | NM_145997 | 0.00042 | -1.33562 |  |
| NM_001017426 // Kdm6b // KDM1 lysine (K)-specific demethylase 6B // 11 B3\|11 // 216850 | Kdm6b | NM_001017426 | 8.44E-07 | -1.35154 |  |
| NM_024245 // Kif23 // kinesin family member 23 // 9 B\|9 // 71819 /// ENSMUST00000034815 | Kif23 | NM_024245 | 1.43E-05 | -1.40956 |  |
| NM_001097621 // Kif26a // kinesin family member 26A // 12 F1\|12 // 668303 /// ENSMUST00 | Kif26a | NM_001097621 | 2.67E-06 | -1.22284 |  |
| NM_172898 // Kirrel2 // kin of IRRE like 2 (Drosophila) // 7 B1\|7 // 243911 /// ENSMUST | Kirrel2 | NM_172898 | 7.19E-06 | -1.41339 |  |
| NM_013598 // Kitl // kit ligand // 10 D1\|10 57.0 cM // 17311 /// ENSMUST00000105283 // | Kitl | NM_013598 | 8.81E-06 | 1.33149 |  |
| NM_172871 // Klhl9 // kelch-like 9 (Drosophila) // 4 C4\|4 // 242521 /// ENSMUST00000094 | Klhl9 | NM_172871 | 0.000482 | -1.30807 |  |
| NM_010114 // Klk1b22 // kallikrein 1-related peptidase b22 // 7 B4\|7 23.07 cM // 13646 | Klk1b22 | NM_010114 | 7.41E-06 | -1.25766 |  |
| NM_008463 // Klra5 // killer cell lectin-like receptor, subfamily A, member 5 // 6 F3\|6 | Klra5 | NM_008463 | 0.000533 | -1.28044 |  |
| NM_013571 // Ksr1 // kinase suppressor of ras 1 // 11 B5\|11 45.78 cM // 16706 /// ENSMU | Ksr1 | NM_013571 | 0.000139 | -1.25784 |  |
| NM_026304 // l7Rn6 // lethal, Chr 7, Rinchik 6 // 7 47.1 cM\|7 E1 // 67669 /// ENSMUST00 | l7Rn6 | NM_026304 | 2.25E-05 | 1.31731 |  |
| NM_010681 // Lama4 // laminin, alpha 4 // 10 B1\|10 25.0 cM // 16775 /// ENSMUST00000019 | Lama4 | NM_010681 | 0.000293 | -1.25015 |  |
| NM_173012 // Letm2 // leucine zipper-EF-hand containing transmembrane protein 2 // 8 A2 | Letm2 | NM_173012 | 2.25E-05 | -1.28295 |  |
| NM_001081298 // Lphn2 // latrophilin 2 // 3 H3\|3 // 99633 /// ENSMUST00000106127 // Lph (adhesion G protein-coupled receptor L2) | Lphn2 (ADGRL2) | NM_001081298 | 0.000353 | -1.32003 |  |
| NM_027452 // Lrfn2 // leucine rich repeat and fibronectin type III domain containing 2 | Lrfn2 | NM_027452 | 0.000666 | -1.23407 |  |
| NM_028886 // Lrguk // leucine-rich repeats and guanylate kinase domain containing // 6\| | Lrguk | NM_028886 | 3.17E-07 | -1.32424 |  |
| NM_001142728 // Lrrc9 // leucine rich repeat containing 9 // 12 C3\|12 // 78257 /// NM_0 | Lrrc9 | NM_001142728 | 1.84E-05 | -1.40843 |  |
| NM_145503 // Lzts2 // leucine zipper, putative tumor suppressor 2 // 19 C3\|19 // 226154 | Lzts2 | NM_145503 | 3.11E-07 | -1.44066 |  |
| NM_001025577 // Maf // avian musculoaponeurotic fibrosarcoma (v-maf) AS42 oncogene homo | Maf | NM_001025577 | 0.000266 | -1.29804 |  |
| NM_011945 // Map3k1 // mitogen-activated protein kinase kinase kinase 1 // 13 D2.2\|13 6 | Map3k1 | NM_011945 | 6.88E-06 | -1.50265 |  |
| NM_001081292 // Map3k10 // mitogen-activated protein kinase kinase kinase 10 // 7 A3\|7 | Map3k10 | NM_001081292 | 0.001024 | -1.25262 |  |
| NM_008279 // Map4k1 // mitogen-activated protein kinase kinase kinase kinase 1 // 7 B1\| | Map4k1 | NM_008279 | 0.000262 | -1.21321 |  |
| BC132343 // Mars2 // methionine-tRNA synthetase 2 (mitochondrial) // 1 C1.2\|1 // 212679 | Mars2 | BC132343 | 6.31E-05 | -1.61519 |  |
| NM_025979 // Mastl // microtubule associated serine/threonine kinase-like // 2 A3\|2 // | Mastl | NM_025979 | 2.18E-05 | -1.4321 |  |
| NM_013595 // Mbd3 // methyl-CpG binding domain protein 3 // 10 C1\|10 43.0 cM // 17192 / | Mbd3 | NM_013595 | 0.000396 | -1.41281 |  |
| NM_175088 // Mdfic // MyoD family inhibitor domain containing // 6 A1\|6 // 16543 /// EN | Mdfic | NM_175088 | 3.32E-06 | -1.47653 |  |
| NM_001081392 // Mdn1 // midasin homolog (yeast) // 4 A5\|4 11.4 cM // 100019 /// ENSMUST | Mdn1 | NM_001081392 | 5.92E-07 | -1.3772 |  |
| NM_023397 // Mdp1 // magnesium-dependent phosphatase 1 // 14 C3\|14 // 67881 /// NR_0283 | Mdp1 | NM_023397 | 0.000456 | -1.37296 |  |
| NM_181407 // Me3 // malic enzyme 3, NADP(+)-dependent, mitochondrial // 7 E1\|7 // 10926 | Me3 | NM_181407 | 6.18E-06 | -1.32625 |  |
| NM_025297 // Mecr // mitochondrial trans-2-enoyl-CoA reductase // 4 D2.3\|4 // 26922 /// | Mecr | NM_025297 | 0.000137 | -1.38716 |  |
| ENSMUST00000001455 // Mef2d // myocyte enhancer factor 2D // 3 F1\|3 43.0 cM // 17261 // | Mef2d | ENSMUST00000001455 | 0.000332 | -1.26374 |  |
| NR_003633 // Meg3 // maternally expressed 3 // 12 F1\|12 54.0 cM // 17263 /// NR_027651 | Meg3 | NR_003633 | 0.00087 | -1.21837 |  |
| NM_026197 // Mett10d // methyltransferase 10 domain containing // 11\|11 B4 // 67493 /// | Mett10d | NM_026197 | 2.71E-05 | -1.33781 |  |
| NR_029790 // Mir100 // microRNA 100 // 9\|9 // 723892 | Mir100 | NR_029790 | 7.34E-07 | -1.37461 |  |
| NR_029566 // Mir10b // microRNA 10b // 2\|2 // 387144 | Mir10b | NR_029566 | 2.45E-06 | -1.41932 |  |
| NR_029541 // Mir126 // microRNA 126 // 2\|2 // 387145 | Mir126 | NR_029541 | 0.000206 | -1.24357 |  |
| NR_029569 // Mir182 // microRNA 182 // 6\|6 // 387177 | Mir182 | NR_029569 | 7.54E-05 | -1.2597 |  |
| NR_029580 // Mir194-1 // microRNA 194-1 // 1\|1 // 387189 | Mir194-1 | NR_029580 | 0.00047 | 1.50094 |  |
| NR_029590 // Mir203 // microRNA 203 // 12\|12 // 387199 | Mir203 | NR_029590 | 0.000181 | -1.31563 |  |
| NR_029593 // Mir206 // microRNA 206 // 1\|1 // 387202 | Mir206 | NR_029593 | 0.00078 | -1.4051 |  |
| NR_029799 // Mir218-2 // microRNA 218-2 // 11\|11 // 723924 | Mir218-2 | NR_029799 | 4.83E-05 | -1.22218 |  |
| NR_029807 // Mir222 // microRNA 222 // X\|X // 723828 | Mir222 | NR_029807 | 0.000138 | -1.34732 |  |
| NR_029880 // Mir379 // microRNA 379 // 12\|12 // 723858 | Mir379 | NR_029880 | 2.58E-07 | -1.57678 |  |
| NR_029971 // Mir451 // microRNA 451 // 11\|11 // 723870 | Mir451 | NR_029971 | 0.000691 | -1.32899 |  |
| NR_030497 // Mir717 // microRNA 717 // X\|X // 751531 | Mir717 | NR_030497 | 8.57E-06 | -1.25846 |  |
| NM_021461 // Mknk1 // MAP kinase-interacting serine/threonine kinase 1 // 4 D1\|4 // 173 | Mknk1 | NM_021461 | 0.000674 | -1.20603 |  |
| NM_001039543 // Mlf1 // myeloid leukemia factor 1 // 3 E1\|3 31.0 cM // 17349 /// NM_010 | Mlf1 | NM_001039543 | 7.60E-06 | -1.53837 |  |
| NM_025962 // Mmachc // methylmalonic aciduria cblC type, with homocystinuria // 4\|4 C7 | Mmachc | NM_025962 | 0.000326 | -1.22223 |  |
| NM_133839 // Mmadhc // methylmalonic aciduria (cobalamin deficiency) cblD type, with ho | Mmadhc | NM_133839 | 0.000478 | -1.27112 |  |
| NM_032007 // Mmp1b // matrix metallopeptidase 1b (interstitial collagenase) // 9 A1\|9 / | Mmp1b | NM_032007 | 0.000637 | -1.24481 |  |
| NM_008610 // Mmp2 // matrix metallopeptidase 2 // 8 C5\|8 44.0 cM // 17390 /// ENSMUST00 | Mmp2 | NM_008610 | 0.000968 | -1.303 |  |
| XM_003086592 // Mrgprb13 // MAS-related GPR, member B13 // 7 B4\|7 // 620137 | Mrgprb13 | XM_003086592 | 1.04E-05 | -1.62267 |  |
| NM_175531 // Mrgprb2 // MAS-related GPR, member B2 // 7 B4\|7 // 243979 /// ENSMUST00000 | Mrgprb2 | NM_175531 | 1.22E-05 | -1.42667 |  |
| NM_026788 // Mthfd2l // methylenetetrahydrofolate dehydrogenase (NADP+ dependent) 2-lik | Mthfd2l | NM_026788 | 0.00065 | -1.47196 |  |
| NM_026829 // Mthfs // 5, 10-methenyltetrahydrofolate synthetase // 9 E3.1\|9 // 107885 / | Mthfs | NM_026829 | 0.000314 | -1.37329 |  |
| NM_001081128 // Mtr // 5-methyltetrahydrofolate-homocysteine methyltransferase // 13 A1 | Mtr | NM_001081128 | 0.000972 | -1.28734 |  |
| NM_008709 // Mycn // v-myc myelocytomatosis viral related oncogene, neuroblastoma deriv | Mycn | NM_008709 | 3.21E-05 | -1.34673 |  |
| NM_026370 // Myst1 // MYST histone acetyltransferase 1 // 7 F3\|7 57.0 cM // 67773 /// N | Myst1 | NM_026370 | 0.000877 | -1.35818 |  |
| NM_001093775 // Myt1l // myelin transcription factor 1-like // 12 A2\|12 14.0 cM // 1793 | Myt1l | NM_001093775 | 1.05E-06 | -1.27399 |  |
| NM_001037098 // Nacc2 // nucleus accumbens associated 2, BEN and BTB (POZ) domain conta | Nacc2 | NM_001037098 | 0.0003 | -1.24376 |  |
| NM_010872 // Naip2 // NLR family, apoptosis inhibitory protein 2 // 13 D1\|13 54.0 cM // | Naip2 | NM_010872 | 4.92E-05 | -1.44678 |  |
| NM_021432 // Nap1l5 // nucleosome assembly protein 1-like 5 // 6\|6 C1 // 58243 /// ENSM | Nap1l5 | NM_021432 | 5.82E-06 | -1.21213 |  |
| NM_008675 // Nbl1 // neuroblastoma, suppression of tumorigenicity 1 // 4 D3\|4 70.0 cM / | Nbl1 | NM_008675 | 5.23E-05 | -1.47134 |  |
| NM_013752 // Nbn // nibrin // 4\|4 A // 27354 /// ENSMUST00000029879 // Nbn // nibrin // | Nbn | NM_013752 | 0.000438 | -1.33125 |  |
| NM_001082476 // Ndor1 // NADPH dependent diflavin oxidoreductase 1 // 2 A3\|2 // 78797 / | Ndor1 | NM_001082476 | 1.23E-05 | -1.43922 |  |
| NM_001114386 // Nedd4l // neural precursor cell expressed, developmentally down-regulat | Nedd4l | NM_001114386 | 2.18E-06 | -1.20322 |  |
| NM_019671 // Net1 // neuroepithelial cell transforming gene 1 // 13 A1\|13 // 56349 /// | Net1 | NM_019671 | 1.42E-05 | -1.51892 |  |
| NM_001164109 // Nfatc1 // nuclear factor of activated T-cells, cytoplasmic, calcineurin | Nfatc1 | NM_001164109 | 5.69E-06 | -1.25902 |  |
| NM_153578 // Nipa1 // non imprinted in Prader-Willi/Angelman syndrome 1 homolog (human) | Nipa1 | NM_153578 | 0.000354 | -1.21931 |  |
| NM_025937 // Nkap // NFKB activating protein // X A3.3 // 67050 /// ENSMUST00000016553 | Nkap | NM_025937 | 0.000712 | -1.20756 |  |
| NM_025719 // Nkapl // NFKB activating protein-like // 13 A3.1\|13 // 66707 /// ENSMUST00 | Nkapl | NM_025719 | 0.000555 | -1.43122 |  |
| NM_138666 // Nlgn1 // neuroligin 1 // 3 A3\|3 // 192167 /// ENSMUST00000099182 // A83009 | Nlgn1 | NM_138666 | 8.78E-06 | -1.25538 |  |
| NM_010928 // Notch2 // Notch gene homolog 2 (Drosophila) // 3 F2.2\|3 45.6 cM // 18129 / | Notch2 | NM_010928 | 0.000106 | -1.23945 |  |
| NM_013780 // Npas3 // neuronal PAS domain protein 3 // 12 C1\|12 // 27386 /// ENSMUST000 | Npas3 | NM_013780 | 9.10E-06 | -1.32184 |  |
| NM_023409 // Npc2 // Niemann Pick type C2 // 12 D1\|12 // 67963 /// ENSMUST00000021668 / | Npc2 | NM_023409 | 0.000145 | -1.27418 |  |
| NM_001177511 // Npffr1 // neuropeptide FF receptor 1 // 10 B4\|10 // 237362 /// ENSMUST0 | Npffr1 | NM_001177511 | 0.000227 | -1.29921 |  |
| NM_001164035 // Ntf3 // neurotrophin 3 // 6 F3\|6 61.0 cM // 18205 /// NM_001164034 // N | Ntf3 | NM_001164035 | 2.56E-05 | -1.58364 |  |
| NM_133501 // Ntng2 // netrin G2 // 2 B\|2 18.0 cM // 171171 /// NM_133500 // Ntng2 // ne | Ntng2 | NM_133501 | 3.50E-05 | -1.24507 |  |
| NM_153092 // Nupl2 // nucleoporin like 2 // 5 A3\|5 // 231042 /// ENSMUST00000049887 // | Nupl2 | NM_153092 | 2.57E-05 | -1.36247 |  |
| NM_177068 // Olfml2b // olfactomedin-like 2B // 1 H3\|1 // 320078 /// ENSMUST00000046792 | Olfml2b | NM_177068 | 1.03E-05 | -1.2566 |  |
| NM_146439 // Olfr937 // olfactory receptor 937 // 9 A5\|9 // 258431 /// ENSMUST000000555 | Olfr937 | NM_146439 | 1.33E-06 | -1.34267 |  |
| NM_146435 // Olfr993 // olfactory receptor 993 // 2 D\|2 // 258427 /// ENSMUST0000009992 | Olfr993 | NM_146435 | 4.84E-07 | -1.41317 |  |
| NM_181848 // Optn // optineurin // 2 A1\|2 0.5 cM // 71648 /// ENSMUST00000114996 // Opt | Optn | NM_181848 | 5.19E-06 | -1.45641 |  |
| ENSMUST00000099933 // P2rx3 // purinergic receptor P2X, ligand-gated ion channel, 3 // | P2rx3 | ENSMUST00000099933 | 0.000294 | -1.27605 |  |
| NM_033321 // P2rx5 // purinergic receptor P2X, ligand-gated ion channel, 5 // 11\|11 B5 | P2rx5 | NM_033321 | 0.000287 | -1.29303 |  |
| NM_008772 // P2ry1 // purinergic receptor P2Y, G-protein coupled 1 // 3 D\|3 // 18441 // | P2ry1 | NM_008772 | 0.000311 | -1.36817 |  |
| NM_019482 // Panx1 // pannexin 1 // 9 A2\|9 // 55991 /// ENSMUST00000056755 // Panx1 // | Panx1 | NM_019482 | 0.000281 | -1.39106 |  |
| NM_172454 // Panx3 // pannexin 3 // 9 A4\|9 // 208098 /// ENSMUST00000011262 // Panx3 // | Panx3 | NM_172454 | 0.00014 | -1.23659 |  |
| NM_198414 // Paqr9 // progestin and adipoQ receptor family member IX // 9 E3.3\|9 // 755 | Paqr9 | NM_198414 | 0.000794 | -1.40283 |  |
| NM_008781 // Pax3 // paired box gene 3 // 1 C4\|1 44.0 cM // 18505 /// NM_001159520 // P | Pax3 | NM_008781 | 4.53E-05 | -1.24034 |  |
| NM_023115 // Pcdh15 // protocadherin 15 // 10 B5.3\|10 40.2 cM // 11994 /// NM_001142735 | Pcdh15 | NM_023115 | 2.80E-07 | -1.32318 |  |
| NM_053136 // Pcdhb11 // protocadherin beta 11 // 18 B3\|18 // 93882 /// ENSMUST000000530 | Pcdhb11 | NM_053136 | 0.000262 | -1.33639 |  |
| NM_053141 // Pcdhb16 // protocadherin beta 16 // 18 B3\|18 // 93887 /// ENSMUST000000514 | Pcdhb16 | NM_053141 | 8.43E-05 | -1.30376 |  |
| NM_053143 // Pcdhb18 // protocadherin beta 18 // 18 B3\|18 // 93889 /// ENSMUST000000559 | Pcdhb18 | NM_053143 | 2.74E-05 | -1.42686 |  |
| NM_053132 // Pcdhb7 // protocadherin beta 7 // 18 B3\|18 // 93878 /// ENSMUST00000053037 | Pcdhb7 | NM_053132 | 6.98E-05 | -1.4686 |  |
| NM_178668 // Pde12 // phosphodiesterase 12 // 14 A3\|14 // 211948 /// ENSMUST00000052932 | Pde12 | NM_178668 | 0.000185 | 1.25242 |  |
| NM_021483 // Pex5l // peroxisomal biogenesis factor 5-like // 3\|3 B // 58869 /// NM_001 | Pex5l | NM_021483 | 2.47E-05 | -1.3026 |  |
| NM_145488 // Pex6 // peroxisomal biogenesis factor 6 // 17 C\|17 // 224824 /// ENSMUST00 | Pex6 | NM_145488 | 4.16E-06 | -1.48653 |  |
| NM_009402 // Pglyrp1 // peptidoglycan recognition protein 1 // 7\|7 A3 // 21946 /// ENSM | Pglyrp1 | NM_009402 | 0.000127 | -1.31142 |  |
| NM_153412 // Phldb2 // pleckstrin homology-like domain, family B, member 2 // 16 B5\|16 | Phldb2 | NM_153412 | 0.000761 | -1.2598 |  |
| NM_025836 // Plin3 // perilipin 3 // 17\|17 D // 66905 /// ENSMUST00000019726 // Plin3 / | Plin3 | NM_025836 | 1.10E-05 | -1.23165 |  |
| NM_001030305 // Pmp2 // peripheral myelin protein 2 // 3 A1\|3 3.9 cM // 18857 /// ENSMU | Pmp2 | NM_001030305 | 9.62E-05 | -1.30189 |  |
| NM_027002 // Polr2d // polymerase (RNA) II (DNA directed) polypeptide D // 18\|18 B2 // | Polr2d | NM_027002 | 0.000201 | -1.56565 |  |
| NM_008895 // Pomc // pro-opiomelanocortin-alpha // 12 A1.1\|12 4.0 cM // 18976 /// ENSMU | Pomc | NM_008895 | 2.10E-05 | -1.56916 |  |
| NM_011134 // Pon1 // paraoxonase 1 // 6 A2\|6 0.5 cM // 18979 /// ENSMUST00000002663 // | Pon1 | NM_011134 | 1.31E-05 | -1.24965 |  |
| NM_001081307 // Ppp1r12b // protein phosphatase 1, regulatory (inhibitor) subunit 12B / | Ppp1r12b | NM_001081307 | 0.000738 | -1.2873 |  |
| NM_016764 // Prdx4 // peroxiredoxin 4 // X F3\|X 67.7 cM // 53381 /// ENSMUST00000026328 | Prdx4 | NM_016764 | 4.68E-05 | -1.39036 |  |
| NM_029355 // Prl7b1 // prolactin family 7, subfamily b, member 1 // 13 A3.1\|13 // 75596 | Prl7b1 | NM_029355 | 1.34E-05 | -1.27736 |  |
| NM_011120 // Prl7d1 // prolactin family 7, subfamily d, member 1 // 13 A3.1\|13 // 18814 | Prl7d1 | NM_011120 | 5.47E-06 | -1.22356 |  |
| NM_011169 // Prlr // prolactin receptor // 15 A1\|15 4.6 cM // 19116 /// ENSMUST00000124 | Prlr | NM_011169 | 0.000145 | -1.57132 |  |
| NM_031499 // Prp2 // proline rich protein 2 // 6 G1\|6 // 83380 /// NM_031499 // Prp2 // | Prp2 | NM_031499 | 6.80E-05 | 3.30407 |  |
| NM_001077363 // Ptbp1 // polypyrimidine tract binding protein 1 // 10 C1\|10 43.0 cM // | Ptbp1 | NM_001077363 | 2.86E-05 | 1.60622 |  |
| NM_133783 // Ptges2 // prostaglandin E synthase 2 // 2 B\|2 // 96979 /// ENSMUST00000028 | Ptges2 | NM_133783 | 0.000968 | -1.28345 |  |
| NM_019766 // Ptges3 // prostaglandin E synthase 3 (cytosolic) // 10 D3\|10 // 56351 /// | Ptges3 | NM_019766 | 1.05E-06 | 1.67832 |  |
| NM_020623 // Pth // parathyroid hormone // 7 F\|7 52.5 cM // 19226 /// ENSMUST0000007979 | Pth | NM_020623 | 0.000349 | -1.20353 |  |
| NM_011207 // Ptpn3 // protein tyrosine phosphatase, non-receptor type 3 // 4 B3\|4 27.0 | Ptpn3 | NM_011207 | 0.000397 | -1.24649 |  |
| NM_009000 // Rab24 // RAB24, member RAS oncogene family // 13 B1\|13 36.0 cM // 19336 // | Rab24 | NM_009000 | 0.000309 | -1.2622 |  |
| NM_011232 // Rad1 // RAD1 homolog (S. pombe) // 15\|15 A2 // 19355 /// ENSMUST0000002285 | Rad1 | NM_011232 | 2.83E-05 | -1.55269 |  |
| NM_178702 // Radil // Ras association and DIL domains // 5 G2\|5 // 231858 /// ENSMUST00 | Radil | NM_178702 | 0.000286 | -1.34649 |  |
| NM_009391 // Ran // RAN, member RAS oncogene family // 5 G1.3\|5 // 19384 /// BC083356 / | Ran | NM_009391 | 0.000222 | -1.25836 |  |
| NM_011243 // Rarb // retinoic acid receptor, beta // 14 1.5 cM\|14 A1-A3 // 218772 /// E | Rarb | NM_011243 | 3.51E-07 | -1.44323 |  |
| NM_027852 // Rarres2 // retinoic acid receptor responder (tazarotene induced) 2 // 6 B2 | Rarres2 | NM_027852 | 0.000172 | -1.54115 |  |
| NM_133914 // Rasa4 // RAS p21 protein activator 4 // 5 G2\|5 // 54153 /// NM_001039103 / | Rasa4 | NM_133914 | 1.14E-05 | -1.2373 |  |
| NM_177644 // Rasal2 // RAS protein activator like 2 // 1 H1\|1 // 226525 /// ENSMUST0000 | Rasal2 | NM_177644 | 1.47E-05 | -1.2475 |  |
| NM_019869 // Rbm14 // RNA binding motif protein 14 // 19 A\|19 // 56275 /// ENSMUST00000 | Rbm14 | NM_019869 | 6.62E-07 | -1.41193 |  |
| NM_001081549 // Rcan1 // regulator of calcineurin 1 // 16 C4\|16 62.0 cM // 54720 /// NM | Rcan1 | NM_001081549 | 0.001033 | -1.2749 |  |
| NM_134006 // Rdh5 // retinol dehydrogenase 5 // 10 D3\|10 72.0 cM // 19682 /// ENSMUST00 | Rdh5 | NM_134006 | 0.000818 | -1.31651 |  |
| NM_026446 // Rgs19 // regulator of G-protein signaling 19 // 2 H4\|2 45.0 cM // 56470 // | Rgs19 | NM_026446 | 7.13E-06 | -1.37479 |  |
| NM_001195748 // Rgs22 // regulator of G-protein signalling 22 // 15 B3.1\|15 // 626596 / | Rgs22 | NM_001195748 | 1.45E-06 | -1.47113 |  |
| NM_019566 // Rhog // ras homolog gene family, member G // 7\|7 F1 // 56212 /// ENSMUST00 | Rhog | NM_019566 | 0.000806 | -1.48011 |  |
| NM_145999 // Rhot2 // ras homolog gene family, member T2 // 17 A3.3\|17 11.6 cM // 21495 | Rhot2 | NM_145999 | 1.77E-05 | -1.23138 |  |
| NM_053271 // Rims2 // regulating synaptic membrane exocytosis 2 // 15 C\|15 16.0 cM // 1 | Rims2 | NM_053271 | 0.000841 | -1.22451 |  |
| NM_182929 // Rims3 // regulating synaptic membrane exocytosis 3 // 4 D2.2\|4 // 242662 / | Rims3 | NM_182929 | 5.61E-05 | -1.23236 |  |
| ENSMUST00000131035 // Rnf213 // ring finger protein 213 // 11 E2\|11 75.0 cM // 672511 | Rnf213 | ENSMUST00000131035 | 5.69E-05 | -1.36853 |  |
| NM_001099632 // Rnf39 // ring finger protein 39 // 17 B1\|17 // 386454 /// ENSMUST000000 | Rnf39 | NM_001099632 | 2.90E-05 | -1.30447 |  |
| NM_172998 // Rnft2 // ring finger protein, transmembrane 2 // 5 F\|5 // 269695 /// NM_00 | Rnft2 | NM_172998 | 0.000622 | -1.34394 |  |
| NM_183263 // Rnmtl1 // RNA methyltransferase like 1 // 11 B5\|11 // 67390 /// ENSMUST000 | Rnmtl1 | NM_183263 | 1.40E-05 | -1.3753 |  |
| NM_025963 // Rps10 // ribosomal protein S10 // 17\|17 B1 // 67097 /// ENSMUST00000114882 | Rps10 | NM_025963 | 9.00E-08 | -1.28735 |  |
| NM_011029 // Rpsa // ribosomal protein SA // 9 F4\|9 71.0 cM // 16785 /// AF140348 // Rp | Rpsa | NM_011029 | 4.47E-05 | -1.35891 |  |
| NM_028898 // Rptor // regulatory associated protein of MTOR, complex 1 // 11 E2\|11 // 7 | Rptor | NM_028898 | 0.000597 | -1.26562 |  |
| NM_011306 // Rxrb // retinoid X receptor beta // 17 B1\|17 18.49 cM // 20182 /// BC04977 | Rxrb | NM_011306 | 0.00036 | -1.40636 |  |
| NM_026535 // Serpina12 // serine (or cysteine) peptidase inhibitor, clade A (alpha-1 an | Serpina12 | NM_026535 | 4.30E-07 | -1.26394 |  |
| NM_001035123 // Setd6 // SET domain containing 6 // 8 D1\|8 // 66083 /// ENSMUST00000034 | Setd6 | NM_001035123 | 4.57E-06 | -1.5067 |  |
| NM_133220 // Sgk3 // serum/glucocorticoid regulated kinase 3 // 1 A2\|1 7.5 cM // 170755 | Sgk3 | NM_133220 | 3.25E-05 | -1.26358 |  |
| NM_028943 // Sgms2 // sphingomyelin synthase 2 // 3 G3\|3 // 74442 /// ENSMUST0000009024 | Sgms2 | NM_028943 | 3.17E-05 | -1.39517 |  |
| NM_011382 // Six4 // sine oculis-related homeobox 4 homolog (Drosophila) // 12 C3\|12 // | Six4 | NM_011382 | 1.94E-06 | -1.25906 |  |
| NM_144539 // Slamf7 // SLAM family member 7 // 1\|1 H2 // 75345 /// ENSMUST00000062219 / | Slamf7 | NM_144539 | 2.94E-05 | -1.24901 |  |
| NM_029415 // Slc10a6 // solute carrier family 10 (sodium/bile acid cotransporter family | Slc10a6 | NM_029415 | 4.94E-05 | -1.21175 |  |
| NM_133649 // Slc12a6 // solute carrier family 12, member 6 // 2 E3\|2 // 107723 /// NM_1 | Slc12a6 | NM_133649 | 0.000141 | -1.28194 |  |
| NM_153081 // Slc16a11 // solute carrier family 16 (monocarboxylic acid transporters), m | Slc16a11 | NM_153081 | 1.66E-07 | -1.49945 |  |
| NM_001029842 // Slc16a6 // solute carrier family 16 (monocarboxylic acid transporters), | Slc16a6 | NM_001029842 | 6.34E-06 | -1.44662 |  |
| NM_146118 // Slc25a25 // solute carrier family 25 (mitochondrial carrier, phosphate car | Slc25a25 | NM_146118 | 0.000797 | -1.35135 |  |
| NM_026331 // Slc25a37 // solute carrier family 25, member 37 // 14 D2\|14 // 67712 /// E | Slc25a37 | NM_026331 | 0.000915 | -1.28863 |  |
| NM_022880 // Slc29a1 // solute carrier family 29 (nucleoside transporters), member 1 // | Slc29a1 | NM_022880 | 0.000263 | -1.27595 |  |
| NM_019741 // Slc2a5 // solute carrier family 2 (facilitated glucose transporter), membe | Slc2a5 | NM_019741 | 0.000604 | -1.34292 |  |
| NM_001033286 // Slc30a10 // solute carrier family 30, member 10 // 1 H5\|1 // 226781 /// | Slc30a10 | NM_001033286 | 8.47E-05 | -1.34682 |  |
| NM_001135149 // Slc39a8 // solute carrier family 39 (metal ion transporter), member 8 / | Slc39a8 | NM_001135149 | 3.02E-05 | -1.24387 |  |
| NM_198106 // Slc9a10 // solute carrier family 9, member 10 // 16 B5\|16 // 208169 /// EN | Slc9a10 | NM_198106 | 0.000123 | -1.31427 |  |
| NM_172429 // Smndc1 // survival motor neuron domain containing 1 // 19 D2\|19 // 76479 / | Smndc1 | NM_172429 | 0.000245 | -1.21821 |  |
| NM_009213 // Smpd2 // sphingomyelin phosphodiesterase 2, neutral // 10 B2\|10 // 20598 / | Smpd2 | NM_009213 | 0.000719 | -1.3464 |  |
| NR_034046 // Snora61 // small nucleolar RNA, H/ACA box 61 // 4\|4 // 100217440 | Snora61 | NR_034046 | 0.000377 | -1.3383 |  |
| AF357427 // Snord115 // Small nucleolar RNA, C/D Box 115 cluster // --- // 493919 | Snord115 | AF357427 | 0.000218 | 1.71172 |  |
| NR_028563 // Snord96a // small nucleolar RNA, C/D box 96A // 11\|11 // 100216534 | Snord96a | NR_028563 | 0.000696 | -1.25629 |  |
| NM_030889 // Sorcs2 // sortilin-related VPS10 domain containing receptor 2 // 5 B3\|5 // | Sorcs2 | NM_030889 | 0.000203 | -1.26567 |  |
| NM_009233 // Sox1 // SRY-box containing gene 1 // 8 A1-A2\|8 4.0 cM // 20664 /// ENSMUST | Sox1 | NM_009233 | 0.000595 | -1.56837 |  |
| NM_011896 // Spry1 // sprouty homolog 1 (Drosophila) // 3 B\|3 // 24063 /// AF176903 // | Spry1 | NM_011896 | 1.40E-05 | -1.49688 |  |
| NM_013539 // Spsb2 // splA/ryanodine receptor domain and SOCS box containing 2 // 6 F2\| | Spsb2 | NM_013539 | 2.64E-06 | -1.37064 |  |
| NM_018873 // Srcin1 // SRC kinase signaling inhibitor 1 // 11 D\|11 // 56013 /// ENSMUST | Srcin1 | NM_018873 | 1.62E-05 | -1.28576 |  |
| NM_029210 // Sv2c // synaptic vesicle glycoprotein 2c // 13 D1\|13 // 75209 /// ENSMUST0 | Sv2c | NM_029210 | 2.87E-05 | -1.34324 |  |
| NM_019796 // Syncrip // synaptotagmin binding, cytoplasmic RNA interacting protein // 9 | Syncrip | NM_019796 | 4.48E-06 | -1.23696 |  |
| NM_001079686 // Syne1 // synaptic nuclear envelope 1 // 10 A1\|10 // 64009 /// NM_153399 | Syne1 | NM_001079686 | 0.000779 | -1.31679 |  |
| XM_985548 // Syngap1 // synaptic Ras GTPase activating protein 1 homolog (rat) // 17 A3 | Syngap1 | XM_985548 | 0.000305 | -1.25089 |  |
| NM_207708 // Syngr1 // synaptogyrin 1 // 15 E1\|15 // 20972 /// NM_009303 // Syngr1 // s | Syngr1 | NM_207708 | 0.000839 | -1.22548 |  |
| NM_198170 // Szt2 // seizure threshold 2 // 4 D2.1\|4 // 230676 /// ENSMUST00000075406 / | Szt2 | NM_198170 | 0.000402 | -1.23724 |  |
| NM_198294 // Tanc1 // tetratricopeptide repeat, ankyrin repeat and coiled-coil containi | Tanc1 | NM_198294 | 1.64E-05 | -1.3927 |  |
| NM_021562 // Tas2r140 // taste receptor, type 2, member 140 // 6G1\|6 63.6 cM // 387616 | Tas2r140 | NM_021562 | 0.00091 | -1.38642 |  |
| NM_032005 // Tbx19 // T-box 19 // 1 H2\|1 86.6 cM // 83993 /// ENSMUST00000027859 // Tbx | Tbx19 | NM_032005 | 4.73E-06 | -1.27196 |  |
| NM_011540 // Tcap // titin-cap // 11 D\|11 // 21393 /// ENSMUST00000008021 // Tcap // ti | Tcap | NM_011540 | 2.47E-06 | -1.22089 |  |
| NM_011567 // Tead4 // TEA domain family member 4 // 6 F3\|6 61.3 cM // 21679 /// NM_0010 | Tead4 | NM_011567 | 8.63E-08 | -1.34308 |  |
| NM_009365 // Tgfb1i1 // transforming growth factor beta 1 induced transcript 1 // 7\|7 F | Tgfb1i1 | NM_009365 | 0.000323 | -1.29596 |  |
| NM_153109 // Tgif2lx1 // TGFB-induced factor homeobox 2-like, X-linked 1 // X E1\|X // 2 | Tgif2lx1 | NM_153109 | 1.01E-05 | -1.39376 |  |
| NM_011579 // Tgtp1 // T-cell specific GTPase 1 // 11 B1.2\|11 // 21822 /// NM_001145164 | Tgtp1 | NM_011579 | 0.000175 | -1.25445 |  |
| NM_146153 // Thrap3 // thyroid hormone receptor associated protein 3 // 4 D2.2\|4 // 230 | Thrap3 | NM_146153 | 8.88E-05 | -1.60216 |  |
| NM_011590 // Timm17a // translocase of inner mitochondrial membrane 17a // 1\|1 F // 218 | Timm17a | NM_011590 | 1.49E-05 | -1.34517 |  |
| NM_011602 // Tln1 // talin 1 // 4 B1\|4 // 21894 /// ENSMUST00000030187 // Tln1 // talin | Tln1 | NM_011602 | 1.97E-05 | -1.24663 |  |
| NM_030682 // Tlr1 // toll-like receptor 1 // 5 C3.1\|5 37.0 cM // 21897 /// ENSMUST00000 | Tlr1 | NM_030682 | 5.20E-06 | -1.46947 |  |
| NM_001025106 // Tmem201 // transmembrane protein 201 // 4 E2\|4 76.4 cM // 230917 /// NM | Tmem201 | NM_001025106 | 1.81E-05 | -1.37898 |  |
| NM_001033321 // Tmem231 // transmembrane protein 231 // 8 E1\|8 // 234740 /// ENSMUST000 | Tmem231 | NM_001033321 | 2.46E-05 | -1.36726 |  |
| NM_009425 // Tnfsf10 // tumor necrosis factor (ligand) superfamily, member 10 // 3 A3\|3 | Tnfsf10 | NM_009425 | 0.000702 | -1.36739 |  |
| NM_033622 // Tnfsf13b // tumor necrosis factor (ligand) superfamily, member 13b // 8 A1 | Tnfsf13b | NM_033622 | 0.000559 | -1.23036 |  |
| NM_172913 // Tox3 // TOX high mobility group box family member 3 // 8 C4\|8 40.0 cM // 2 | Tox3 | NM_172913 | 1.98E-05 | -1.22095 |  |
| NM_028718 // Traf3ip1 // TRAF3 interacting protein 1 // 1 D\|1 // 74019 /// ENSMUST00000 | Traf3ip1 | NM_028718 | 6.63E-06 | -1.41759 |  |
| NM_026508 // Trap1 // TNF receptor-associated protein 1 // 16 A1\|16 1.8 cM // 68015 /// | Trap1 | NM_026508 | 0.000383 | -1.35199 |  |
| NM_029726 // Trdn // triadin // 10 A4\|10 // 76757 | Trdn | NM_029726 | 4.97E-05 | -2.20764 |  |
| NM_133975 // Trip12 // thyroid hormone receptor interactor 12 // 1 F\|1 // 14897 /// ENS | Trip12 | NM_133975 | 0.000547 | -1.21714 |  |
| NM_011639 // Trip6 // thyroid hormone receptor interactor 6 // 5 G2\|5 // 22051 /// ENSM | Trip6 | NM_011639 | 7.73E-05 | -1.52453 |  |
| NM_026642 // Trmt12 // tRNA methyltranferase 12 homolog (S. cerevisiae) // 15 D1\|15 // | Trmt12 | NM_026642 | 3.98E-06 | -1.28043 |  |
| NM_013735 // Trp53bp1 // transformation related protein 53 binding protein 1 // 2 E5\|2 | Trp53bp1 | NM_013735 | 5.58E-05 | -1.25069 |  |
| NM_015767 // Ttpa // tocopherol (alpha) transfer protein // 4 A3\|4 22.7 cM // 50500 /// | Ttpa | NM_015767 | 0.000184 | -1.27299 |  |
| NM_011657 // Tulp3 // tubby-like protein 3 // 6 F3\|6 62.5 cM // 22158 /// ENSMUST000000 | Tulp3 | NM_011657 | 2.27E-06 | -1.65923 |  |
| NM_018793 // Tyk2 // tyrosine kinase 2 // 9 A3\|9 // 54721 /// ENSMUST00000001036 // Tyk | Tyk2 | NM_018793 | 0.000195 | -1.27557 |  |
| NM_021288 // Tyms // thymidylate synthase // 5 B1\|5 18.2 cM // 22171 /// NR_033402 // T | Tyms | NM_021288 | 6.85E-06 | -1.56865 |  |
| BC025894 // Ubc // ubiquitin C // 5 G1.1\|5 64.0 cM // 22190 /// ENSMUST00000100700 // G | Ubc | BC025894 | 0.000335 | -1.33968 |  |
| NM_145500 // Ubtd1 // ubiquitin domain containing 1 // 19 C3\|19 // 226122 /// ENSMUST00 | Ubtd1 | NM_145500 | 1.07E-05 | -1.30234 |  |
| NM_013918 // Usp25 // ubiquitin specific peptidase 25 // 16 C3.1\|16 // 30940 /// ENSMUS | Usp25 | NM_013918 | 0.00022 | -1.2463 |  |
| NM_011682 // Utrn // utrophin // 10 A1\|10 3.0 cM // 22288 /// ENSMUST00000076817 // Utr | Utrn | NM_011682 | 4.69E-07 | -1.26496 |  |
| NM_020505 // Vav3 // vav 3 oncogene // 3 G1\|3 // 57257 /// NM_146139 // Vav3 // vav 3 o | Vav3 | NM_020505 | 1.14E-05 | -1.63395 |  |
| NM_009504 // Vdr // vitamin D receptor // 15 F1\|15 // 22337 /// ENSMUST00000023119 // V | Vdr | NM_009504 | 5.46E-05 | -1.22443 |  |
| NM_009506 // Vegfc // vascular endothelial growth factor C // 8\|8 B3 // 22341 /// ENSMU | Vegfc | NM_009506 | 1.35E-06 | -1.54424 |  |
| NM_145842 // Vmn1r69 // vomeronasal 1 receptor 69 // 7 A1\|7 // 252904 /// ENSMUST000000 | Vmn1r69 | NM_145842 | 1.73E-05 | -1.33171 |  |
| NM_001104626 // Vmn2r15 // vomeronasal 2, receptor 15 // 5 F\|5 // 211223 | Vmn2r15 | NM_001104626 | 2.36E-05 | -1.43643 |  |
| NM_001102580 // Vmn2r76 // vomeronasal 2, receptor 76 // 7 D3\|7 // 675969 | Vmn2r76 | NM_001102580 | 0.000155 | -1.3381 |  |
| XM_003086154 // Vwa3b // von Willebrand factor A domain containing 3B // 1 B\|1 // 70853 | Vwa3b | XM_003086154 | 7.61E-06 | -1.33925 |  |
| NM_033572 // Wbscr16 // Williams-Beuren syndrome chromosome region 16 homolog (human) / | Wbscr16 | NM_033572 | 0.000225 | -1.36379 |  |
| NM_172598 // Wdhd1 // WD repeat and HMG-box DNA binding protein 1 // 14 C1\|14 // 218973 | Wdhd1 | NM_172598 | 1.70E-05 | -1.36683 |  |
| NM_009522 // Wnt3a // wingless-related MMTV integration site 3A // 11 B1.3\|11 32.0 cM / | Wnt3a | NM_009522 | 0.000384 | -1.24597 |  |
| NM_207212 // Wtip // WT1-interacting protein // 7 B1\|7 // 101543 /// ENSMUST00000038537 | Wtip | NM_207212 | 0.000399 | 1.26029 |  |
| NM_133216 // Xpnpep1 // X-prolyl aminopeptidase (aminopeptidase P) 1, soluble // 19 D2\| | Xpnpep1 | NM_133216 | 0.000298 | -1.25968 |  |
| NM_009533 // Xrcc5 // X-ray repair complementing defective repair in Chinese hamster ce | Xrcc5 | NM_009533 | 0.000167 | -1.28052 |  |
| NM_026858 // Xrcc6bp1 // XRCC6 binding protein 1 // 10 D3\|10 // 68876 /// NM_001159559 | Xrcc6bp1 | NM_026858 | 0.000407 | -1.35917 |  |
| NM_001145930 // Yeats2 // YEATS domain containing 2 // 16 A3\|16 // 208146 /// NM_001033 | Yeats2 | NM_001145930 | 0.000708 | -1.25435 |  |
| NM_001163013 // Ythdc2 // YTH domain containing 2 // 18 B3\|18 // 240255 /// BC138263 // | Ythdc2 | NM_001163013 | 0.000509 | -1.22792 |  |
| NM_177660 // Zbtb10 // zinc finger and BTB domain containing 10 // 3 A1\|3 // 229055 /// | Zbtb10 | NM_177660 | 0.000168 | -1.30433 |  |
| NM_172643 // Zbtb41 // zinc finger and BTB domain containing 41 homolog // 1 F\|1 // 226 | Zbtb41 | NM_172643 | 0.00096 | -1.31863 |  |
| NM_011762 // Zfp59 // zinc finger protein 59 // 7 A3 // 22717 /// ENSMUST00000108332 // | Zfp59 | NM_011762 | 0.00041 | -1.20108 |  |
| NM_011763 // Zfp9 // zinc finger protein 9 // 6 F1 // 22750 /// ENSMUST00000112838 // Z | Zfp9 | NM_011763 | 0.003488 | -1.29285 |  |
