# Supplementary material for: Synaptic and mitochondrial mechanisms behind alcohol-induced imbalance of excitatory/inhibitory synaptic activity and associated cognitive and behavioral abnormalities
Source: Transl Psychiatry. 2024 Jan 22;14:51. doi: 10.1038/s41398-024-02748-8 (PMC10803756; doi:10.1038/s41398-024-02748-8)
Supplement: Supplementary file 3 — Table S2 [file 41398_2024_2748_MOESM3_ESM.docx]

| **Table S2. The developmental ethanol exposure-induced dysregulated synaptic genes in P60 mouse brains that are associated with psychological disorders and cognitive dysfunction** | |
| --- | --- |
| **Genes** | **Diseases or Functions Annotation** |
| Acute agitation | CHRM1,HTR1A,HTR1D |
| Acute mania | CHRM1,HTR1A,HTR1D |
| Agitation | CHRM1,CNR1,HTR1A,HTR1D |
| Alzheimer disease | ADGRL2,APOE,CHRM1,CNR1,GRIN2B,GRIN2C,GRIN2D,GRIN3B,HTR1A,HTT |
| Amyotrophic lateral sclerosis | APOE,CHRM1,GRIN2B,GRIN2C,GRIN2D,GRIN3B,HTR1A,SYNE1 |
| Anxiety Disorders | CHRM1,CNR1,GRIK1,GRIN2B,GRIN2C,GRIN2D,GRIN3B,HTR1A,HTR1D,HTT,SLC29A1 |
| Asperger syndrome | GRIN2B,GRIN2C,GRIN2D,GRIN3B |
| Autism | CHRM1,DMD,GRIN2B,GRIN2C,GRIN2D,GRIN3B,HTR1A,NLGN1,PCDH15,SLC29A1 |
| Behavioral deficit | APOE,CHRM1,CNR1,GRIN2B,GRIN2C,GRIN2D,GRIN3B,HTR1A,HTT,KCNH1,SYNGAP1 |
| Bipolar depression | CHRM1,GRIN2B,GRIN2C,GRIN2D,GRIN3B,HTR1A,HTR1D |
| Bipolar disorder | CACNA1C,CHRM1,GRID1,GRIK1,GRIN2B,GRIN2C,GRIN2D,GRIN3B,GRM4,HTR1A,HTR1D,SYNE1 |
| Bipolar II disorder | GRIN2B,GRIN2C,GRIN2D,GRIN3B,HTR1A,HTR1D |
| Bipolar spectrum disorder | CHRM1,GRIN2B,GRIN2C,GRIN2D,GRIN3B,HTR1A,HTR1D |
| Brain damage | CACNA1C,CHRM1,CNR1,GRIK1,GRIN2B,GRIN2C,GRIN2D,GRIN3B,HTR1A,HTR1D,HTT |
| Cerebral degeneration | APOE,CHRM1,GRIN2B,GRIN2C,GRIN2D,GRIN3B,HTR1A,HTT,SYNE1 |
| Delirium | CHRM1,GRIN2B,HTR1A,HTR1D |
| Delusional disorder | GRIN2B,GRIN2C,GRIN2D,GRIN3B,HTR1A,HTR1D |
| Dementia | ADGRL2,APOE,CACNA1C,CHRM1,CNR1,GRIK1,GRIN2B,GRIN2C,GRIN2D,GRIN3B,HTR1A,HTR1D,HTT |
| Depressive disorder | APOE,CACNA1C,CHRM1,FGA,GRIK1,GRIN2B,GRIN2C,GRIN2D,GRIN3B,HTR1A,HTR1D,HTT,ITGB5,SLC29A1 |
| Disorder of basal ganglia | APOE,CACNA1C,CHRM1,CNR1,GRIN2B,GRIN2C,GRIN2D,GRIN3B,GRM4,HAP1,HTR1A,HTR1D,HTT,RPSA |
| Drug abuse | GRIN2B,HTR1A,HTT |
| Drug dependence | CHRM1,CNR1,GRIK1,GRIN2B,HTR1A,HTR1D,SLC29A1 |
| Eating Disorders | CHRM1,CNR1,GRIK1,GRIN2B,HAP1,HTR1A |
| Familial pervasive developmental disorder | DMD,GRIN2B,NLGN1,NTNG2,PCDH15 |
| Familial psychiatric disease | APOE,CACNA1C,CHRM1,CNR1,DMD,GRIK1,GRIK3,GRIN2B,GRIN2C,GRIN2D,GRIN3B,GRM4,HAP1,HTR1A,HTR1D,HTT,NLGN1,NTNG2,PCDH15,RPSA,SYNE1 |
| Familial schizophrenia | CACNA1C,DMD,GRIK3,GRIN2B |
| First episode psychosis | CHRM1,HTR1A,HTR1D,SLC29A1 |
| Heroin dependence | GRIN2B,HTR1A,SLC29A1 |
| Huntington Disease | APOE,CHRM1,CNR1,GRIN2B,GRIN2C,GRIN2D,GRIN3B,GRM4,HAP1,HTR1D,HTT,RPSA |
| Hyperactive behavior | CHRM1,CNR1,HTR1A,HTT,KCNH1,SYNGAP1 |
| Irritable behavior | CHRM1,GRIN2B,GRIN2C,GRIN2D,GRIN3B,HTR1A |
| Major affective disorder | APOE,CACNA1C,CHRM1,GRID1,GRIK1,GRIN2B,GRIN2C,GRIN2D,GRIN3B,GRM4,HTR1A,HTR1D,ITGB5,SLC29A1,SYNE1 |
| Major depression | APOE,CACNA1C,CHRM1,GRIK1,GRIN2B,GRIN2C,GRIN2D,GRIN3B,HTR1A,HTR1D,ITGB5,SLC29A1 |
| Mania | CACNA1C,CHRM1,HTR1A,HTR1D |
| Manic bipolar I disorder | CHRM1,HTR1A,HTR1D |
| Mixed bipolar I disorder | CHRM1,HTR1A,HTR1D |
| Mental retardation | CACNA1C,CBLN2,CHRM1,DMD,GRIN2B,KCNH1,NTNG2,SLC29A1,SYNE1,SYNGAP1,TANC1 |
| Mood Disorders | APOE,CACNA1C,CHRM1,FGA,GRID1,GRIK1,GRIN2B,GRIN2C,GRIN2D,GRIN3B,GRM4,HTR1A,HTR1D,HTT,ITGB5,SLC29A1,SYNE1 |
| Non-affective psychosis | CHRM1,GRIN2B,GRIN2C,GRIN2D,GRIN3B,HTR1A,HTR1D |
| Obsessive-compulsive disorder | GRIK1,GRIN2B,GRIN2C,GRIN2D,GRIN3B,HTR1A,HTT |
| Obsessive-compulsive spectrum disorder | CHRM1,GRIK1,GRIN2B,GRIN2C,GRIN2D,GRIN3B,HTR1A,HTT |
| Opioid dependence | CNR1,GRIN2B,HTR1A,SLC29A1 |
| Panic disorder | GRIN2B,GRIN2C,GRIN2D,GRIN3B,HTR1A,HTR1D |
| Parkinson's disease | APOE,CACNA1C,CHRM1,CNR1,GRIN2B,GRIN2C,GRIN2D,GRIN3B,HTR1A |
| Pervasive developmental disorder | ADGRL2,CBLN2,CHRM1,DMD,GRIN2B,GRIN2C,GRIN2D,GRIN3B,HTR1A,LRFN2,NLGN1,NTNG2,PCDH15,SLC29A1 |
| Pervasive developmental disorder not otherwise specified | GRIN2B,GRIN2C,GRIN2D,GRIN3B |
| Phobic anxiety disorder | GRIN2B,GRIN2C,GRIN2D,GRIN3B |
| Post-traumatic stress disorder | CHRM1,CNR1,GRIK1,GRIN2B,GRIN2C,GRIN2D,GRIN3B,HTR1A,HTR1D,SLC29A1 |
| Pseudobulbar affect | CHRM1,GRIN2B,GRIN2C,GRIN2D,GRIN3B |
| Psychosis | APOE,CHRM1,GRIN2B,GRIN2C,GRIN2D,GRIN3B,HTR1A,HTR1D,HTT,SLC29A1 |
| Recurrent mood disorder | CHRM1,HTR1A,HTR1D |
| Relapsed schizophrenia | CHRM1,HTR1A,HTR1D |
| Schizoaffective disorder | CHRM1,GRIN2B,GRIN2C,GRIN2D,GRIN3B,HTR1A,HTR1D |
| Schizophrenia | CACNA1C,CHRM1,CNR1,DMD,GRID1,GRIK1,GRIK3,GRIN2B,GRIN2C,GRIN2D,GRIN3B,GRM4,HTR1A,HTR1D,LRFN2,NTNG2,PCDH15,RIMS3 |
| Schizophrenia spectrum disorder | CACNA1C,CHRM1,CNR1,DMD,GRID1,GRIK1,GRIK3,GRIN2B,GRIN2C,GRIN2D,GRIN3B,GRM4,HTR1A,HTR1D,LRFN2,NLGN1,NTNG2,PCDH15,RIMS3,SLC29A1 |
| Severe psychological disorder | APOE,CACNA1C,CHRM1,CNR1,DMD,GRID1,GRIK1,GRIK3,GRIN2B,GRIN2C,GRIN2D,GRIN3B,GRM4,HTR1A,HTR1D,HTT,ITGB5,LRFN2,NTNG2,PCDH15,RIMS3,SLC29A1,SYNE1 |
| Social anxiety disorder | GRIK1,GRIN2B,GRIN2C,GRIN2D,GRIN3B,HTR1A,HTR1D |
| Suicidal ideation | GRIN2B,GRIN2C,GRIN2D,GRIN3B,HTR1A |
| Susceptibility to autism | DMD,NLGN1,PCDH15 |
| Temporal lobe atrophy | CHRM1,GRIN2B,HTT |
| Traumatic brain injury | CHRM1,GRIK1,GRIN2B,GRIN2C,GRIN2D,GRIN3B,HTR1A,HTR1D |
| Treatment resistant depression | CHRM1,GRIN2B,GRIN2C,GRIN2D,GRIN3B,HTR1A |
