# Supplementary material for: Synaptic and mitochondrial mechanisms behind alcohol-induced imbalance of excitatory/inhibitory synaptic activity and associated cognitive and behavioral abnormalities
Source: Transl Psychiatry. 2024 Jan 22;14:51. doi: 10.1038/s41398-024-02748-8 (PMC10803756; doi:10.1038/s41398-024-02748-8)
Supplement: Supplementary file 4 — Table S3 [file 41398_2024_2748_MOESM4_ESM.docx]

| **Table S3. The association of P7 ethanol exposure-induced dysregulated mitochondrial metabolism-related genes in P60 mouse brains with neurological diseases** | |
| --- | --- |
| **Diseases or Functions Annotation** | **Genes** |
| Childhood encephalopathy | GOT2,MECR,SLC29A1,TYMS |
| Syndromic developmental disorder with epilepsy | GOT2,MTHFS,SLC29A1 |
| Syndromic neurodevelopmental disorder | GOT2,KDM6B,MTHFS,SLC29A1 |
| Childhood onset movement disorder | MECR,SLC29A1 |
| Hereditary neuropathy | KDM6B,MARS2,MECR,OPTN |
| Abnormal morphology of retinal pigment epithelium | CASP3,OPTN |
| Loss of RPE cells | OPTN |
| Autosomal recessive spastic ataxia type 3 | MARS2 |
| Amyotrophic lateral sclerosis type 12 | OPTN |
| Neurodevelopmental disorder with coarse facies and mild distal skeletal abnormalities | KDM6B |
| 1p/19q deletion negative anaplastic astrocytoma | TYMS |
| Neurodevelopmental disorder with microcephaly, epilepsy, and hypomyelination | MTHFS |
| Early infantile epileptic encephalopathy type 82 | GOT2 |
| Childhood-onset dystonia with optic atrophy and abnormal basal ganglia | MECR |
| Familial neurodevelopmental disorder | GOT2,KDM6B,MTHFS,SLC29A1 |
| Mild cognitive impairment | CASP3,SLC29A1 |
| Hyperplasia of neurons | CASP3 |
| Familial epilepsy | GOT2,MTHFS,SLC29A1 |
| Epilepsy | GOT2,MTHFS,SLC25A25,SLC29A1 |
| Speech disorder | KDM6B,MTHFS |
| Epilepsy or neurodevelopmental disorder | GOT2,KDM6B,MTHFS,SLC25A25,SLC29A1 |
| 1p/19q co-deletion negative glioma | TYMS |
| Supratentorial glioblastoma | TYMS |
| Autosomal recessive neurological disorder | GOT2,MARS2,MECR,MTHFS,OPTN |
| Autosomal recessive neurodevelopmental syndrome | GOT2,MTHFS |
| Recurrent anaplastic astrocytoma | TYMS |
| Cryptococcal meningitis | TYMS |
| IDH mutation positive astrocytoma | TYMS |
| Ohtahara syndrome | GOT2,SLC29A1 |
| Autosomal recessive amyotrophic lateral sclerosis | OPTN |
| Regression of glioma | CASP3 |
| Advanced glioblastoma | TYMS |
| Cranioschisis | CASP3 |
| Primary central nervous system lymphoma | GOT2,TYMS |
| Refractory epilepsy | SLC29A1 |
| Preclinical stage amyotrophic lateral sclerosis | CASP3 |
| Mobius syndrome | KDM6B |
| Congenital neurological disorder | CASP3,KDM6B,MTHFS,SLC29A1,TYMS |
| Spinocerebellar ataxia | CASP3,MARS2 |
| Familial encephalopathy | CASP3,GOT2,MARS2,MECR,MTHFS,OPTN,SLC29A1 |
